# Comparison and consolidation of microarray data sets of human tissue expression

**DOI:** 10.1186/1471-2164-11-305

**Published:** 2010-05-14

**Authors:** Jenny Russ, Matthias E Futschik

**Affiliations:** 1 Institute for Theoretical Biology, Charité, Humboldt University, Invalidenstrasse 43, 10115 Berlin, Germany; 2 Institute for Biotechnology and Bioengineering (Laboratório Associado), Centre for Molecular and Structural Biomedicine, University of Algarve, 8005-139 Faro, Portugal

## Abstract

**Background:**

Human tissue displays a remarkable diversity in structure and function. To understand how such diversity emerges from the same DNA, systematic measurements of gene expression across different tissues in the human body are essential. Several recent studies addressed this formidable task using microarray technologies. These large tissue expression data sets have provided us an important basis for biomedical research. However, it is well known that microarray data can be compromised by high noise level and various experimental artefacts. Critical comparison of different data sets can help to reveal such errors and to avoid pitfalls in their application.

**Results:**

We present here the first comparison and integration of four freely available tissue expression data sets generated using three different microarray platforms and containing a total of 377 microarray hybridizations. When assessing the tissue expression of genes, we found that the results considerably depend on the chosen data set. Nevertheless, the comparison also revealed statistically significant similarity of gene expression profiles across different platforms. This enabled us to construct consolidated lists of platform-independent tissue-specific genes using a set of complementary measures. Follow-up analyses showed that results based on consolidated data tend to be more reliable.

**Conclusions:**

Our study strongly indicates that the consolidation of the four different tissue expression data sets can increase data quality and can lead to biologically more meaningful results. The provided compendium of platform-independent gene lists should facilitate the identification of novel tissue-specific marker genes.

## Background

Tissue in the human body shows a fascinating variety of structures and functions ranging from simple protection by the epidermis to complex information processing in the cortex. All these different types of tissues have in common that they are highly adapted to their specific task. How such variety can emerge from the same genetic code is not only scientifically an intriguing question, but also of fundamental medical relevance. To address this question, a necessary step is the generation of comprehensive catalogs of gene expression in different tissues.

Microarrays have become highly suitable tools for such an ambitious endeavour as they allow the genome-wide measurement of transcription abundance. Most early gene expression studies of human tissues were disease-orientated, i.e. the expression profiles of pathogenic tissues were compared to corresponding normal tissues. For instance, microarrays have extensively been used to detect previously unrecognized cancer subtypes [1,2] and to predict clinical outcome [3,4]. Yet, it is also crucial to create a comprehensive gene expression atlas for normal human tissues to facilitate rapid identification of new marker genes for improved diagnosis and of target genes for medical interventions. Furthermore, such atlas would undoubtedly increase our general understanding in physiology and would give us new insights into functions of genes. Indeed, large-scale microarray experiments have recently been used to construct such powerful expression repositories [5-8].

However, general concerns about the reliability of microarray data have been raised by several studies showing poor congruence of different microarray platforms [9-12]. Moreover, there are many experimental factors such as array platform, tissue handling, RNA isolation method and hybridization procedure which strongly influence the results of expression studies. As a consequence, results derived using a single microarray experiment may be seriously compromised by experimental bias. In contrast, the use of multiple independent data sets can be beneficial for the data reliability [13,14].

This motivated us to critically compare and to consolidate four major microarray data sets of human tissue expression due to their outstanding importance for functional genomics and large-scale systems biology. Notably, although these data sets have rapidly become popular resources in biomedical research [15-19], this is the first study which systematically compares them. The ultimate aim of our investigations was to construct a compendium of platform-independent gene lists assessing the expression in normal human tissues by various means. Such a compendium can not only dispense biomedical researchers from cumbersome manual querying of multiple data sets, but also help to avoid potential pitfalls caused by platform-dependent artefacts.

The outline of the study was the following: After compilation of the tissue expression data sets, we first examined their overall similarity. For the assessment of tissue specific expression, we generated multiple gene lists utilizing complementary measures to detect distinct features of tissue expression. These lists were subsequently used for a critical comparison of results derived from the different experiments. Finally, we assembled lists of platform-independent tissue-specific genes. The assessment of the consolidated data indicated that they can provide a more reliable basis for the study of tissue expression.

## Results and Discussion

### Compilation of tissue expression from different experiments

In this study, we compared and analyzed four different microarray data sets comprising predominately gene expression of normal human tissues. Detailed information for the data sets is given in Table 1 and in the *Methods *section. The four data sets were produced using three different microarray platforms: Agilent spotted oligonucleotide microarrays (here referred to as Rosetta1 [5] and Rosetta2 [20]), Affymetrix GeneChips (referred to as Geneatlas [8]), and cDNA microarrays (referred to as Stanford [21]). Table 1 shows that the four data sets differ considerably in the number of samples, ranging from 50 to 158, and in the number of analyzed tissues, ranging from 35 to 79.

**Table 1:** Summary of analyzed tissue expression data sets.

Data set	Publication	Technology	Number of genes	Number of samples	Number of tissues
**Rosetta1**	Johnson et al., Science 2003	Agilent oligonucleotide exon microarrays	9,394	50	50
**Rosetta2**	Schadt et al., Genome Biology 2004	Agilent oligonucleotide microarrays	13,367	54	54
**Stanford**	Shyamsundar et al., Genome Biology 2005	Dual-channel cDNA microarrays	13,984	115	35
**Geneatlas**	Su et al., PNAS 2004	Affymetrix HG-U133A and GNF1H arrays	16,499	158	79

The number of genes refers to the probes on the arrays that could be mapped to corresponding Entrez Gene identifiers.

For comparative analysis, the microarray probes were mapped to their corresponding Entrez Gene identifiers to obtain a common index system. Expression values of replicated probes were averaged. To facilitate the comparison, the samples were assigned to 19 main tissue classes based on their physiology and histology. Subsequently, the expression values were averaged over samples belonging to the same tissue class. Further details of the applied data pre-processing and the assignments of samples to tissue classes can be found in the *Methods *section and the Additional file 1 (*Supplementary Materials*).

### Comparison of tissue expression data sets

**Figure 1:**
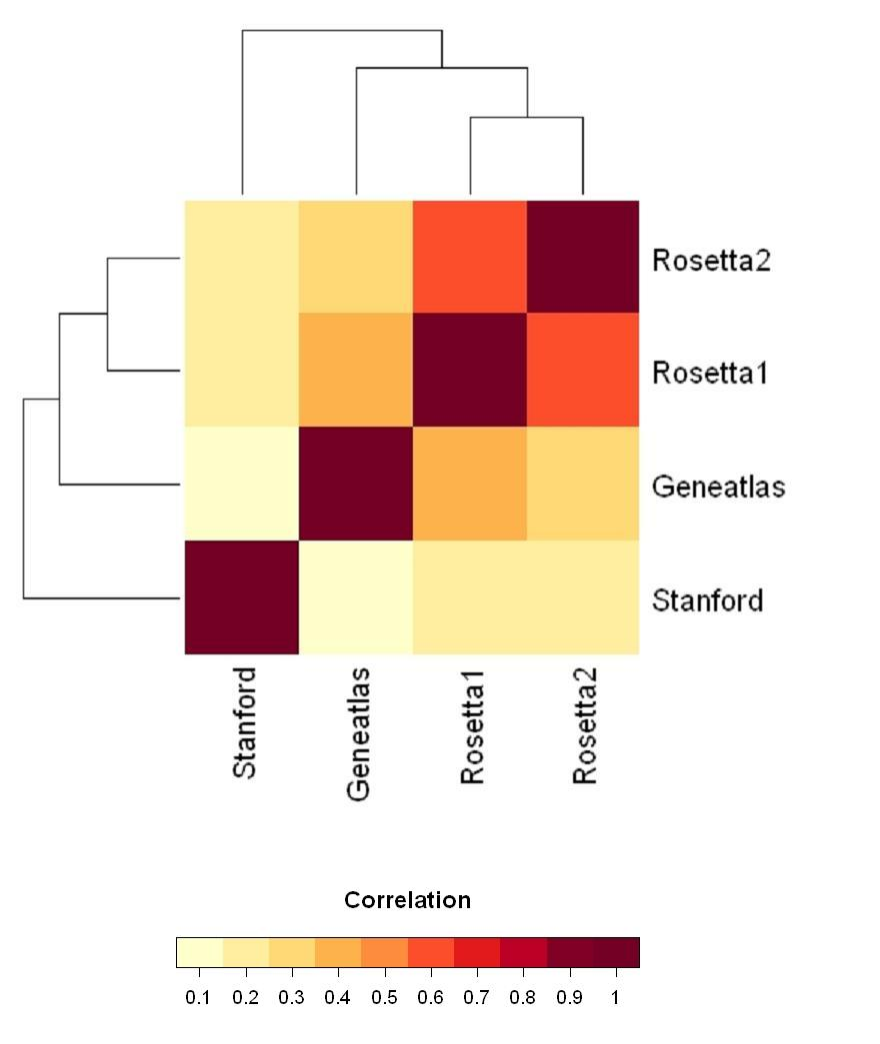
**Cluster image map of gene-based correlation of correlation matrix**. Hierarchical clustering was performed for the assessment of the pair-wise similarities of the data sets. The numerical correlation of correlation values (from Table 2) are represented according to the displayed colour-bar.

First, we examined whether the four different data sets show similar gene expression profiles. This comparison was based on 6685 non-redundant genes that were common to all data sets. To assess their global similarity, we calculated the correlations for all pairs of genes within a data set and correlated them between different data sets. This so-called correlation of correlation measure was previously proposed to examine the overall similarity of expression patterns in two different microarray data sets [22]. Large correlation of correlations indicates similar overall co-expression of genes in two data sets. Their significance was assessed by comparing the results of the corresponding randomized expression matrices. Table 2 displays the results of this analysis. The correlations of correlations of the original data sets are considerably larger than those calculated for random correlation matrices. Thus, all data sets are more similar in their gene expression patterns than expected by chance. However, the observed similarities are not equally distributed across all pairs of data sets. To examine this issue further, relationships between the data sets were analyzed by hierarchical clustering and were visualized as cluster image map in Figure 1. Notably, the data sets Rosetta1 and Rosetta2 show the largest similarity which might have been expected as both are based on the same microarray platform. In contrast, the clustering indicates that the Stanford data set is least similar to the other data sets. Notably, we obtained comparable results when we analyzed the global pair-wise correlation of tissue classes in the four data sets (Additional file 1 - Table S1).

**Table 2:** Gene-wise correlation of correlations.

		Rosetta1	Rosetta2	Geneatlas	Stanford
**Rosetta1**	**Original**	**1**	**0.61**	**0.41**	**0.21**
	Random	0.0026	0.0033	0.0036	0.0044
**Rosetta2**	**Original**	**0.61**	**1**	**0.35**	**0.24**
	Random	0.0033	0.0021	0.0028	0.0037
**Geneatlas**	**Original**	**0.41**	**0.35**	**1**	**0.16**
	Random	0.0036	0.0028	0.0036	0.0051
**Stanford**	**Original**	**0.21**	**0.24**	**0.16**	**1**
	Random	0.0044	0.0037	0.0051	0.0048

Correlation of gene-wise correlations between the four data sets and between corresponding randomized gene expression matrices.

**Figure 2:**
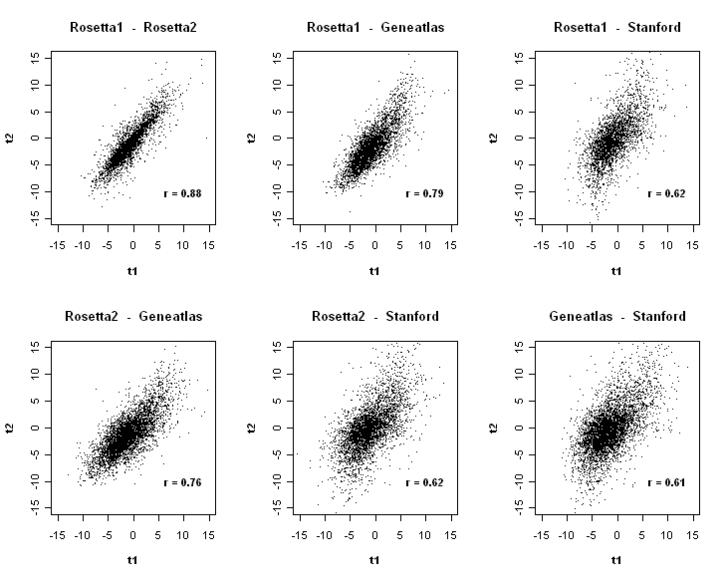
**Comparison of gene expression in brain and non-brain tissues**. Differential expression between brain and non-brain tissues was assessed by performing a gene-wise unpaired Student's t-test. To compare the results from different data sets, t-scores derived from each data set were plotted versus those from the other data sets for the corresponding genes. Additionally, the Pearson correlation coefficient is given.

These results are supported by the analysis of the differential gene expression for specific tissue types. Figure 2 illustrates this comparison for brain-specific expression. The differential expression found in the data sets by Rosetta1 and Rosetta2 were highly correlated whereas the Stanford data set showed more distinct results compared to the other data sets. Nevertheless, a prominent similarity in differential expression across all data sets exists. Statistical assessment of the pair-wise Pearson correlation coefficients demonstrated their high significance (p < 10 ^-16^).

### Assessment of tissue specificity

A main purpose of the four tissue expression data sets has been the identification of tissue-specific and housekeeping genes. Does the observed divergence influence the detection of such genes? To address this question we used two different measures to capture distinct features of tissue-specific expression.

**Figure 3:**
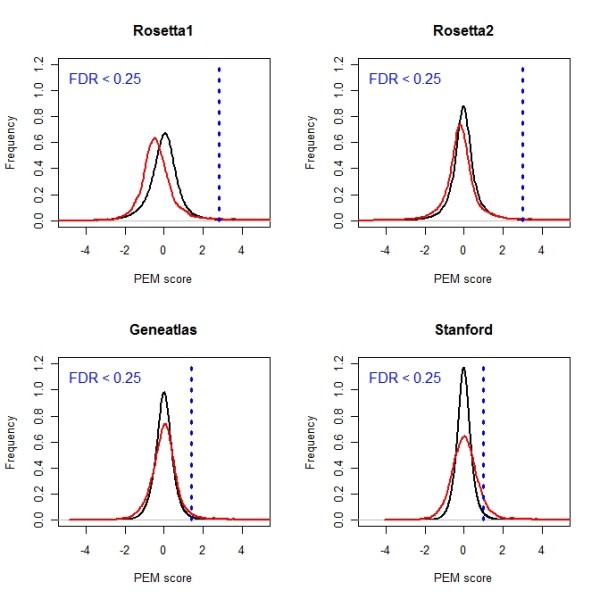
**Distribution of PEM scores for liver tissue**. The displayed distributions (shown in red) are based on the scores calculated for liver tissue in the compared data sets. To determine the significance of PEM scores, background distributions (shown in black) were generated. The threshold for PEM scores corresponding to FDR < 0.25 is shown. The displayed distributions are based on Gaussian kernel estimates.

First, we scrutinized the expression data with respect to tissue-specific over- or under-expression. For this task, we used the Preferential Expression Measure (PEM) originally proposed by Huminiecki and co-workers [13]. The PEM scores the expression of a gene in a given tissue in relation to its average expression in all tissues. It reports a positive value for genes over-expressed and a negative value for genes under-expressed in the specific tissue, respectively. Mathematically, it is defined as the logged (with base 2) ratio of the expression in a chosen tissue and the mean expression in all tissues. Calculation of the PEM score for the different tissue classes and data sets shows that the majority of genes have a score close to zero, as shown in Figure 3 for liver tissue, indicating that only a subset of genes are differentially expressed in tissues. The significance of PEM scores was assessed using a permutation-based procedure. First, we generated background distributions of PEM scores for each tissue class based on repeated randomization of the association between arrays and tissue classes. These distributions represent the set of PEM scores which we would expect to observe by chance for the corresponding tissue class. Subsequently, the observed PEM scores were compared with the generated background distribution and the statistical significance in terms of false discovery rates were calculated. Details of the calculation can be found in the *Methods *section. Since the data distributions differ considerably between the compared microarray experiments, the permutation procedure was applied to each microarray set separately. Figure 3 illustrates the approach displaying the distributions of observed and generated PEM scores for liver tissue. Note that this approach allowed us to compare tissue-specific expression across different microarray experiments.

**Figure 4:**
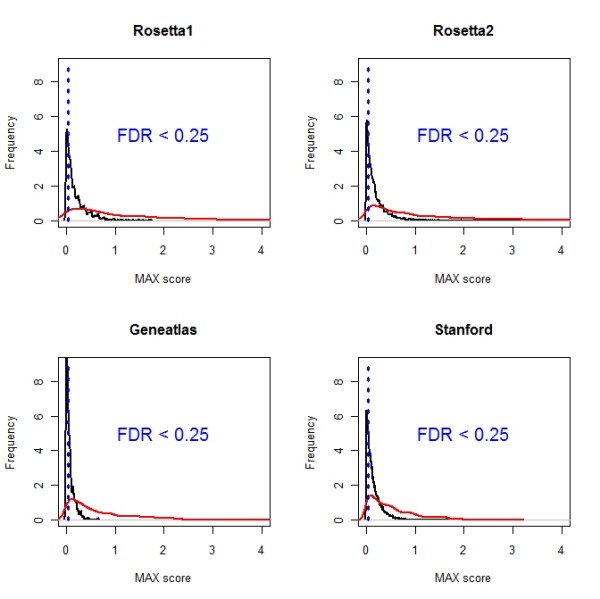
**Distribution of MAX scores for brain tissue**. The displayed distributions (shown in red) are based on the scores derived for brain tissue in compared data sets. To determine the significance of MAX scores, background distributions (shown in black) were generated. The threshold for significant MAX scores obtaining FDR < 0.25 is shown. The displayed distributions are based on Gaussian kernel estimates.

Since PEM is defined with respect to the average expression, a gene can obtain high PEM scores for multiple tissues. However, researchers are frequently interested in genes that are over-expressed in a *unique *tissue type as they can serve as suitable candidates for marker genes. Thus, we introduced an alternative measure which addresses this aspect: First, we determined the tissue for which the gene is maximally over-expressed. Subsequently, the difference between the largest and second largest value for expression was calculated. This difference was termed MAX. A gene with a large MAX value displays high gene expression in one tissue but not in the other tissues, i.e. it is uniquely over-expressed in a single tissue. The corresponding MAX scores for remaining tissues (for which this gene is not maximally expressed) were set to zero. We determined the significance of the MAX scores using the same permutation-based procedure as described for the PEM scores. Figure 4 displays the observed distribution of MAX scores and the generated background distributions derived for brain tissue in the four data sets.

### Identification and comparison of tissue-specific genes

To examine in more detail the similarity of tissue-specific expression in different data sets, we created separate gene lists for each data set and tissue type using the described measures. Genes with positive PEM score and FDR < 0.25 were defined here as tissue-specifically over-expressed and with negative PEM score and FDR < 0.25 as tissue-specifically under-expressed. For assessing the uniqueness of tissue expression, we defined a gene as *uniquely *over-expressed in a given tissue if it is maximal expressed in this tissue and FDR < 0.25 holds for the corresponding MAX score. Note that we have chosen a rather high FDR, but the main conclusions of our comparison also hold for more stringent values (see also Additional file 1 - Figure S1).

**Figure 5:**
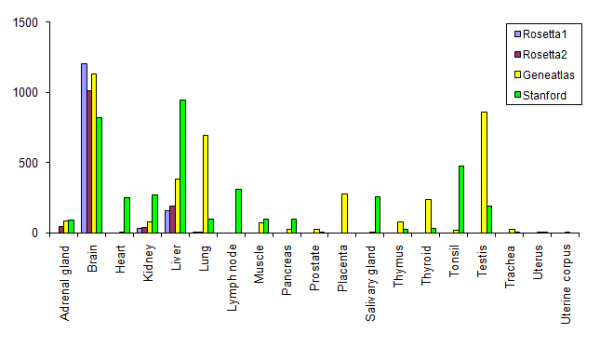
**Number of tissue-specifically over-expressed genes in the single data sets**. For each data set and tissue type, genes were identified as specifically over-expressed if the corresponding PEM score is positive and achieves FDR < 0.25. Note that tissue-specific over-expressed genes could not be identified for several tissue classes in the Rosetta1, Rosetta2 and Stanford data sets due to the missing replicates. Only genes included in all four data sets were considered here.

For gene lists based on the PEM score, we found that the absolute number of over-expressed genes varies greatly between the 19 tissues type (Figure 5). Due to missing or low number of replicates, only for 4 tissue classes (brain, kidney, liver and lung) include significantly over-expressed genes in all 4 data sets. This finding is also reflected in the numbers of tissue-specific genes common to the different data sets, which is generally small (Additional file 1 - Figure S2). For brain tissue, for instance, we found that in average only about 40% of over-expressed genes in one data set are also over-expressed in all the other data sets.

**Figure 6:**
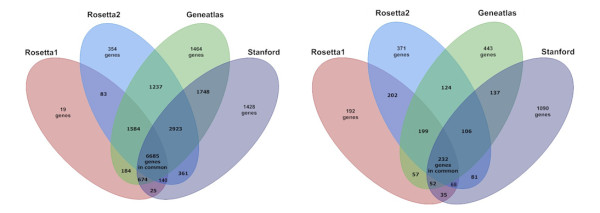
**Concordance of assayed and uniquely over-expressed genes**. Gene lists were derived for all four experiments and examined for common genes. The concordance of all assayed genes in the different microarray experiments is shown on the left side. The obtained concordance of uniquely over-expressed genes (with MAX value > 0 and FDR < 0.25) in adrenal gland, brain, kidney, liver, and lung is depicted on the right side. The largest overlap was detected between Rosetta 1 and Rosetta 2 sharing on average 21% of the detected genes. In contrast, Stanford and Rosetta2 display the smallest overlap sharing only 14% of the detected genes.

A similar divergence in the set of tissue-specific genes was obtained, when we compared the lists of uniquely over-expressed genes which were generated using the MAX measure (Figure 6). Altogether, we detected 3389 genes as uniquely over-expressed in a tissue-specific manner. However, the majority (62%) of those genes were detected in only one data set, whereas about one sixth (15%) was supported by two experiments. The number of genes consistently determined as uniquely over-expressed was strikingly small: Only 232 genes (i.e. 7%) fulfilled the criterion in all datasets.

Notably, the number of genes detected as tissue specific appeared to depend on the numbers of available samples in a tissue class (Additional file 1 - Table S2). Tissue classes with fewer samples seemed to be associated with fewer tissue-specific genes. To elucidate this statistical dependency in more detail, we simulated the effect of a reduction in samples on the detection of tissue-specifically over-expressed genes. Here, we chose the brain tissue class as reference, since it comprised the largest numbers of samples in all four data sets (Additional file 1 - Table S2) and also yielded large numbers of over-expressed genes (Figure 5). Based on random sampling, the number of brain samples in a data set was reduced while the samples in all other tissue classes were conserved. Subsequently, the number of brain specific genes in the modified data set was calculated. Generally, a strong decrease in the number of significant genes was observed for a reduced number of included brain samples confirming the suspected interference. For instance, less than 500 genes were on average classified as brain specific in the Geneatlas data set if only 6 brain samples were included. This contrasts the 1123 genes detected using the full set of 44 brain samples (Additional file 1 - Figure S5).

To enable a less biased comparison between tissues, we sought to reduce the influence of the number of samples on the number of detected genes by a tissue-specific adjustment of the FDR threshold for significance. Again, brain tissue was used for calibration. The underlying idea was to select a modified FDR threshold, so that the number of brain-specific genes found for a reduced data set (i.e. with a smaller number of brain samples) remains the same as for the full data set with the original FDR threshold. As before, we selected randomly brain samples, constructed reduced data sets and calculated the significance of genes. For calibration, the number of selected brain samples was set equal to the number of samples found in the other tissue classes. As result, we observed that the FDR threshold had generally to be increased for tissue classes including fewer samples (Additional file 1 - Table S5). For example, the adjusted FDR threshold of 0.08 for liver tissue, which comprises of 5 samples in the Stanford set, corresponds to a FDR threshold of 0.05 for brain tissue, which comprises of 8 samples in the same data set. For muscle tissue with only 3 samples, the equivalent threshold is considerably larger with FDR < 0.18. Notably, the application of the adjusted FDR thresholds resulted in drastic changes in the distribution of tissue-specially over-expressed genes (Additional file 1 - Figure S7A-D). In particular, tissue classes with few samples showed a strong relative increase in tissue-specific genes.

For our comparison, we utilized the pre-processed data as provided by the authors of the microarray experiments (except for the Stanford data set which was additionally normalized), as most other previous studies employing these resources have also used the readily available pre-processed data and our analyses should be applicable for the interpretation of these studies. However, it is well known that normalization and pre-processing procedure can have considerable influence on the results of microarray data analyses. Also, it has been shown that re-annotation of Affymetrix GeneChips can improve the quality of derived transcriptional data. Thus, we examined whether alternative data pre-processing procedures may improve the concordance of the microarray data sets. For the Geneatlas dataset, we used an alternative annotation and normalization scheme as described in the *Methods *section. The Rosetta1 and Rosetta2 datasets were additionally log-transformed. Finally, between-array normalization was applied to all four data sets. For comparison, we applied the same procedures as for the original datasets, i.e. correlation of correlation and correlation of brain-specific expression, and calculated the overlap for uniquely over-expressed genes. Notably, we did not observe a general increase in the similarity. While the measured correlation values tend to decrease (Additional file 1 - Table S3, Figure S3), the overlap slightly increased (Additional file 1 - Figure S4). Nevertheless, the overlap of all 4 alternatively pre-proceeded data sets remained small with 240 genes compared to 232 for the original data sets. As a general improvement of similarity was not observed using alternative pre- processing and annotation schemes, we chose to use the original data sets for the subsequent analyses. This choice also facilitates the assessment of previous studies which were generally based on the original data sets. Notably, the comparison of alternative processing schemes for the Geneatlas data set revealed a strong similarity of expression changes as well as a highly significant overlap of detected tissue-specific genes (Additional file 1 - Table S4, Figure S5).

### Uniquely over-expressed genes in brain and liver

The conducted comparison revealed a substantial influence of the microarray platform on the composition of the constructed gene lists. As such platform-dependent bias can compromise the data quality; we reasoned that a consolidated list of common tissue-specific genes might yield more reliable results. This list could be simply generated by direct intersection of lists derived from the different experiments. However, Figures 6 and S2 show that such procedure would result in a very limited number of genes. We therefore chose the alternative scheme (described in the following section) to merge the evidence from different data sets.

Genes obtaining large MAX scores in all four data sets should be strong candidates for reliably being tissue-specifically over-expressed. We assessed this hypothesis by examining in detail brain- and liver-specifically over-expressed genes. For integrative scoring, we first ranked the genes in the four data sets according to their MAX values and calculated their mean ranks, which was subsequently used for generated consolidated lists. The follow-up analysis of the resulting gene lists demonstrated that this simple scheme is remarkably efficient for a reliable identification of tissue-specific genes.

For brain tissue, the 20 top-ranking genes were individually inspected and the vast majority proved to be previously known as brain-specifically or preferentially in brain expressed genes (Table 3). Most of the genes were important structural proteins in neuronal and glial cells or involved in neuronal signalling. The top-ranking gene encodes one of the major filament proteins in astrocytes, the glial fibrillary acidic protein (GFAP). This protein is already employed as a maker for astrocytes and, in its mutated form, is associated with Alexander disease, a rare fatal neurodegenerative disease [23]. Although many of the top-scoring genes obtained a high rank in all data sets, there were notable exceptions. Myelin basic protein, ranked 14^th ^in the consolidated list, is a crucial protein for the myelination of axons in the central nervous systems. In contrast, it was ranked relatively low (as 100^th^) in the Stanford dataset. Similarly, enolase 2 is a neuron-specific enzyme in the glycolytic pathway and is used as marker [24]. In the consolidated list, it was ranked 26^th^, whereas it was ranked considerably lower in two datasets (Rosetta1: 133, Stanford: 111). This suggests that *bona fide *markers can be detected by consolidation despite having a less prominent position in single data sets.

**Table 3:** Top 20 brain-specific genes.

Entrez GeneID	Consolidated Rank	Rosetta1 MAX	Rosetta2 MAX	Stanford MAX	Genealtas MAX	Locuslink Symbol	Description
2670	1	5.25	4.90	5.02	3.48	GFAP	Glial fibrillary acidic protein, a major intermediate filament proteins of astrocytes
11075	2	4.57	4.45	4.53	5.04	STMN2	Stathmin-like 2, a neuronal growth-associated protein
2596	3	6.38	5.69	4.16	2.75	GAP43	Growth associated protein 43, regulates growth of axons during development and regeneration
4747	4	4.86	3.47	3.33	3.75	NEFL	Neurofilament, light polypeptide, a major constituent of the axoskeleton
5354	5	5.13	2.72	4.30	4.45	PLP1	Proteolipid protein 1, predominant myelin protein present in CNS
5375	6	4.47	4.95	2.97	2.09	PMP2	Peripheral myelin protein 2
9568	7	3.41	3.89	4.25	2.19	GABBR2	Gamma-aminobutyric acid (GABA) B receptor 2
9118	8	4.06	4.02	3.24	1.79	INA	Internexin neuronal intermediate filament protein alpha
1759	9	3.24	3.35	2.93	2.69	DNM1	Dynamin 1, involved in clathrin-mediated endocytosis
6456	10	3.57	3.45	2.46	2.51	SH3GL2	SH3-domain GRB2-like 2, Endophilin 1, mediator of synaptic vesicle formation
6616	11	5.54	3.25	1.52	4.17	SNAP25	Synaptosomal-associated protein 25 kDa, a SNARE protein required for neuronal exocytosis
29114	12	4.10	3.09	3.80	2.00	NP22	Neural protein 22
4155	13	5.10	3.46	1.30	4.79	MBP	Myelin basic protein, major constituent of myelin sheath of oligodendrocytes and Schwann
11076	14	2.67	3.43	2.84	2.27	TPPP	Tubulin polymerization promoting protein
6285	15	2.87	2.21	3.54	3.64	S100B	S100 calcium binding protein B, glial-derived protein serving as neurotrophic factor and neuronal survival protein
4741	16	2.97	2.78	2.55	2.52	NEFM	Neurofilament, medium polypeptide 150 kDa
1463	17	3.25	5.22	1.54	1.97	NCAN	Neurocan, involved in the modulation of cell adhesion and migration.
3797	18	3.86	2.98	2.56	1.56	KIF3C	Neurospecific KIF3C kinesin family member 3
29106	19	4.83	2.81	2.50	1.51	SCG3	Secretogranin III, a neuroendocrine secretory protein
81551	20	3.44	2.41	1.85	2.67	STMN4	Stathmin-like 4, regulation of the microtubule cytoskeleton

The top twenty genes based on the integrative MAX scoring are displayed. Besides the consolidated rank, the MAX values for the single data sets are also presented.

For liver tissue, the top-ranking genes are listed in Table 4. Closer inspection supports the conjecture that the integrative scoring can achieve successful identification of tissue-specific genes. Many of the 20 top genes encode for plasma proteins known to be synthesized by the liver. An example is the top-scoring gene, hemopexin that encodes for a plasma protein that binds to heme with high affinity and serves as a diagnostic feature of hemolytic anemia [25]. Other highly ranked genes correspond to liver-specific enzymes involved in lipid synthesis (e.g. FABP1) or drug metabolism (e.g. Cytochrome P450 enzymes). Altogether, the composition of the integrated gene list reflects well the multiple functions of the liver. Therefore, we may conclude that the performed consolidation can provide tissue-specific gene lists of high confidence.

**Table 4:** Top 20 liver-specific genes.

Entrez GeneID	Consolidated Rank	Rosetta1 MAX	Rosetta2 MAX	Stanford MAX	Genealtas MAX	Locuslink Symbol	Description
3263	1	5.25	4.90	5.02	3.48	HPX	Hemopexin, heme-binding plasma protein synthesized by the liver
3053	2	4.57	4.45	4.53	5.04	SERPIND1	Serpin peptidase inhibitor, clade D, member 1, cofactor of heparin in plasma
6580	3	6.38	5.69	4.16	2.75	SLC22A1	Solute carrier family 22 member 1, main organic cation uptake system in hepatocyte
462	4	4.86	3.47	3.33	3.75	SERPINC1	Serpin peptidase inhibitor, clade C (antithrombin), member 1
8608	5	5.13	2.72	4.30	4.45	RDH16	Retinol dehydrogenase 16, involved in lipid metabolism in liver
344	6	4.47	4.95	2.97	2.09	APOC2	Apolipoprotein C-II, component of very low density lipoprotein
1571	7	3.41	3.89	4.25	2.19	CYP2E1	Cytochrome P450, family 2, subfamily E, polypeptide 1. cytochrome oxidase system
6906	8	4.06	4.02	3.24	1.79	SERPINA7	Serpin peptidase inhibitor, clade A (antitrypsin), member 7
1559	9	3.24	3.35	2.93	2.69	CYP2C9	Cytochrome P450, family 2, subfamily 2, polypeptide 9 -
1551	10	3.57	3.45	2.46	2.51	CYP3A7	Cytochrome P450, family 3, subfamily A, polypeptide 7
732	11	5.54	3.25	1.52	4.17	C8B	Complement component 8, beta polypeptide
731	12	4.10	3.09	3.80	2.00	C8A	Complement component 8, alpha polypeptide
7448	13	5.10	3.46	1.30	4.79	VTN	Vitronectin - plasma protein promoting cell adhesion
350	14	2.67	3.43	2.84	2.27	APOH	Apolipoprotein H (beta-2-glycoprotein I)
1373	15	2.87	2.21	3.54	3.64	CPS1	CPS1 carbamoyl-phosphate synthetase 1, enzyme in the hepatic urea cycle
1361	16	2.97	2.78	2.55	2.52	CPB2	Carboxypeptidase B2 plasma protein regulating fibrinolyses,
3273	17	3.25	5.22	1.54	1.97	HRG	Histidine-rich glycoprotein, plasma protein
338	18	3.86	2.98	2.56	1.56	APOB	Apolipoprotein B, isoform apoB-100, exclusively synthesized in the liver
2168	19	4.83	2.81	2.50	1.51	FABP1	Fatty acid binding protein 1 found in the liver
10998	20	3.44	2.41	1.85	2.67	SLC27A5	Solute carrier family 27 (fatty acid transporter), member 5, involved in lipid synthesis

The top twenty genes based on the integrative MAX scoring are displayed.

### Assessment of reliability of derived gene lists

The inspection of the derived consolidated lists for brain and liver indicated that multiple confirmations led to accurate identification of tissue-specific genes. Since the comparison of tissue-specific genes pointed to a considerable platform dependency, another positive aspect of the proposed integration method would be a reduced dependency, or respectively, an increased reliability of the detected gene lists. Clearly, the derived consolidated gene lists show reduced platform dependence. But is this simply the consequence that the MAX scores from all compared platforms were merged, or can the proposed integration method still reduce the platform dependency of gene lists when compared to data which were not included in the integration? To assess whether the latter is the case, we conducted a cross-validation. We examined whether the consolidated MAX scores for brain tissue derived from three integrated data sets show a higher correlation with the MAX scores of the fourth data set than with the MAX scores of the three individual data sets. This procedure was performed for all four microarray experiments.

**Figure 7:**
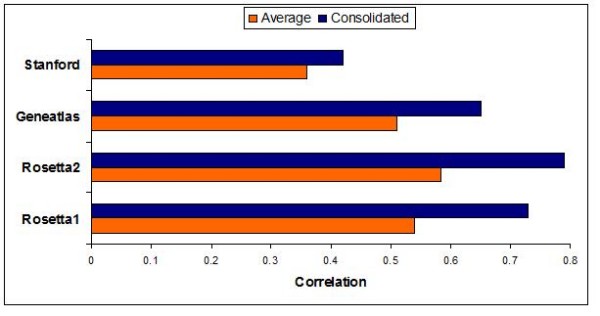
**Spearman correlation of MAX scores for brain tissue**. To assess the reliability of the consolidated gene lists we performed a cross validation. The number of genes in each data set was reduced to the genes found in all four data sets with positive MAX score in brain tissue. The diagram shows the average Spearman correlation of each data set vs. the other data sets and of three consolidated data sets vs. the data set that was left out.

Strikingly, the consolidated gene lists showed considerable larger correlation with the independent gene list than expected based on the observed correlation between the single data sets (Figure 7). For instance, the average Spearman correlation of MAX scores for brain tissue between the Geneatlas data set and the remaining sets is 0.51. In contrast, the corresponding correlation with the integrated MAX score derived from the other three data sets is 0.65. Similar results were observed for the other data sets in the cross-validation demonstrating that the proposed integration can indeed lead to increased reliability of the deducted tissue-specific gene lists.

### Gene ontology analysis of single data sets and integrated data

**Figure 8:**
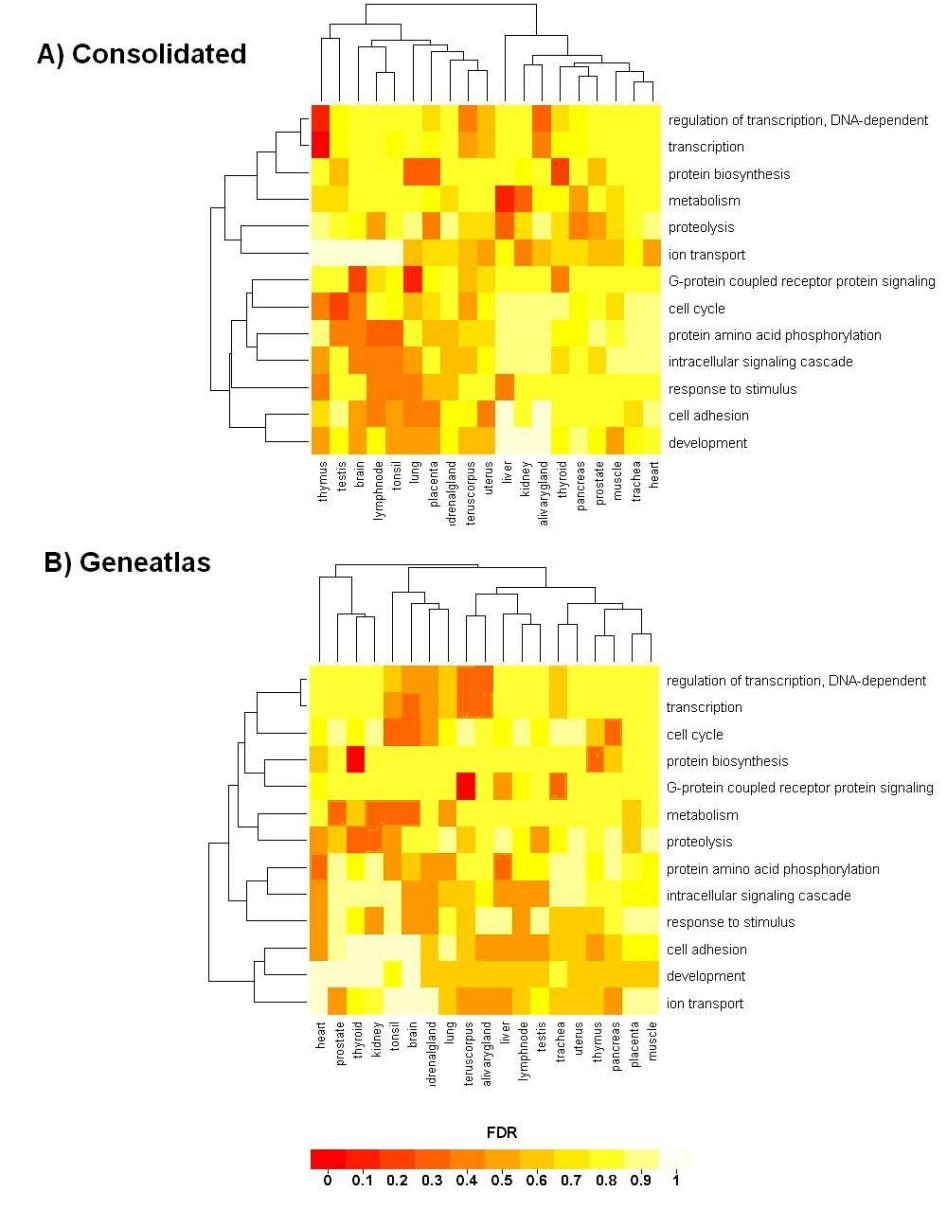
**Cluster image map for the GO analysis of the consolidated gene lists and of the Geneatlas data set**. Genes of the consolidated lists and of the lists derived solely from the Geneatlas data were mapped to the biological processes to which the genes are assigned in Gene Ontology (GO). The significance of enrichment in informative GO categories was derived by using Fisher's exact test and adjusted for multiple testing. Hierarchical clustering was subsequently performed based on the derived false discovery rates (FDR). The cluster image maps display the FDR of the GO enrichment according to the colour-bar at the bottom.

Besides enhancing the data reliability, do the consolidated gene lists also improve the functional characterization of tissue types? To address this question, each tissue type was represented by their corresponding specifically expressed genes. Subsequently, these lists were associated to biological processes described in Gene ontology (GO). Using Fisher's exact test, the significance of enrichment of the over-expressed genes in a biological process was derived for each tissue type. To facilitate its evaluation, we restricted the GO analysis to 18 so-called informative GO categories. For functional characterization, tissues types and GO categories were hierarchically clustered based on the significance of enrichment as similarity measure. This functional characterization allowed us to compare the reliability of the consolidated gene list with the gene lists obtained from single data sets. In Figure 8, the results are displayed for the consolidated gene lists and contrasted to the clustering obtained if only one data set (e.g. Geneatlas here) was used.

Assessment of the results showed that we obtained biologically more reasonable results for consolidated lists. For example, pancreas tissue was strongly associated with the GO category "proteolysis", which corresponds to its exocrine function. Similarly, liver tissue was strongly associated with "metabolism", which is known to be the most prominent function of the liver (Figure 8A). In contrast, we do not obtain such a strong association for the gene lists based solely on the Geneatlas data set (Figure 8B). Moreover, tissues based predominantly (or to a high grade) on muscle cells such as (skeletal) muscle, heart (i.e. cardiac muscle) and trachea (which contains smooth muscular fibres) form a cluster based on the consolidated gene lists. Likewise, the lymphoid tissues, i.e. lymph node and tonsil, showed a similar GO annotation profile and were grouped together. This was not the case for the clustering based on tissue-specific genes that were derived using Geneatlas data only. Notably, similar short-comings were observed for corresponding clustering analyses of the other three single data sets (Additional file 1 - Figure S8). These findings support the conjecture that the consolidated gene lists lead to more reliable and biologically more meaningful results.

## Conclusions

Microarrays have become widely used tools to measure gene expression for the prediction of disease outcome, the profiling of new disease subtypes and the identification of disease-specific markers [1-4]. To achieve these goals, it is crucial to acquire detailed knowledge about expression levels in normal tissue. Several microarray studies have therefore aimed to profile human tissue expression and have rapidly become important resources for biomedical investigations [15-19]. However, the vast majority of the subsequent analyses (including our own [16]) have relied on only one of these data sets. Any bias inflicted by the chosen data set may severely compromise the reliability of results. Due to their increasing importance, we re-analysed four large tissue expression data sets. Our aim was to examine whether a platform-dependency is prominent or rather neglectable compared to the biological variability monitored in these data sets.

Remarkably, we found that the detected tissue-specific expression is strongly influenced by the choice of microarray platform. The observed divergence could have multiple reasons. Foremost, the set of tissue samples was not the same for the compared experiments. Thus, differences would be caused by biological variation. Furthermore, experimental factors such as difference in probe sequences, RNA handling, hybridization protocols, scanning, and image analysis are likely to contribute to the observed divergence. In our study, we applied some alternative pre-processing schemes to improve the concurrency of the data sets. Although these schemes did not yield a larger overlap of tissue-specific genes, we anticipate that improved pre-processing procedures can support the consolidation of the data generated by different microarray data sets. Especially, a more accurate annotation of probes (i.e. their mapping of corresponding genes) will help to improve the comparability of microarray platforms [26,27]. Thus, ongoing efforts in this direction remain important for the re-utilization of existing microarray data.

Despite the differences, however, all four data sets show a larger similarity than expected by chance. This similarity provided us the basis for the consolidation of the different expression experiments. In fact, dissimilarity of expression patterns can be used to eliminate spurious expression patterns. It has recently been demonstrated that the expression of genes displaying a similar pattern for different platforms tends to have an increased reproducibility with respect to external validation by qRT-PCR [28]. Thus, cross-platform comparison can be employed as powerful means to eliminate unreliable gene expression measurements.

Therefore, we propose the integration of multiple tissue expression data sets. We generated lists of tissue-specific genes for each single data set and integrated them to a single consolidated list. Naturally, data integration would not be beneficial if any of the data sets to be integrated is unreliable. However, the re-analyses as well as the original studies strongly demonstrated that each of the data sets shows convincible overall reproducibility. Nevertheless, we observed biologically more accurate results for the integrated list in contrast to lists derived by one experiment only in an exemplary GO analysis. Additionally, we constructed a compendium including tissue expression data for 18909 non-redundant genes and scores for tissue specificity to allow researchers their own analysis. The consolidation is based on simple, but yet effective scoring methods. We would like to note that the rather stringent requirements can be relaxed using the compendium. For example, we would find a considerably larger overlap of tissue-specific genes if we only require that they are shared between two or three data sets. Therefore, researchers using the compendium are encouraged to adjust the thresholds to their own requirements. To facilitate such further analysis our compendium provides the complete data sets with the specificity scores. The compendium will be particularly beneficial for analyses involving a large number of genes. A possible application might be the construction of tissue-specific networks which have attracted increased attention in systems biology [29,30]. Using the compiled data, potential platform-dependency can easily be eliminated in order to avoid severe artefacts in the analyses.

In conclusion: Although various studies have compared disease-related microarray experiments, this analysis constitutes the first systematic comparison of large-scale microarray data sets of normal human tissues. We detected a prominent platform bias, which however can be overcome by data integration. Thus, an important contribution of this study is in the compilation of platform-independent tissue-specific lists which will be provided freely. We anticipate that they will be of great asset for biomedical scientists. Finally, we hope that our study provides a solid basis for the future wide-spread use of microarray tissue expression data in genomic research.

## Methods

### Data collection and pre-processing

The data sets Rosetta1 [5] and Rosetta2 [20] were provided directly by the authors. Both data sets were generated using Agilent spotted oligonucleotide microarrays. The provided data sets comprised expression patterns for 10,000 and 50,000 transcripts in 50 and 54 different tissues, respectively. The data sets provided have been background corrected (due to spatial variation and sequence-specific effects) and normalized using dye-swap normalization (i.e. based on reverse labelling of target and reference samples) by the authors. For the assessment of the influence of preprocessing on the comparison, we additionally log-transformed and subsequently normalized the data across arrays using the quantile normalization which is based on the transformation of the original value to the corresponding quantile's value of a reference chip [31]

Shyamsundar *et al*. [21] measured gene expression in 115 tissue samples on dual-channel cDNA microarrays containing 39,711 human cDNAs. The raw data was downloaded from the Stanford Microarray Database. We normalized the raw data using optimized local intensity-dependent normalization (OLIN) which is based on iterative local regression and optimization of model parameters by internal cross-validation. It has been demonstrated that it can correct favourably for potential dye and spatial biases in two-channel microarray data [32,33]. Optionally, quantile normalization was applied.

Su *et al*. [8] created a gene expression profile of 79 human tissues using Affymetrix HG-U133A and customized GNF1H arrays comprising 45,953 probe sets. Altogether, 16499 non-redundant genes were profiled by replicated hybridizations.

Expression summaries had been derived using the Affymetrix Microarray Suite 5 (MAS5) algorithm with global median scaling. The MAS5-processed data were downloaded from the Geneatlas webpage. To improve the concordance with the other data sets, we also calculated expression levels using an alternative annotation and normalization scheme. Starting from the CEL files (downloaded from NCBI GEO repository - Series GSE1133), we used an customized CDF annotation file for the U133A GeneChip (downloaded from http://www.xlab.unimo.it/GA_CDF/) and the GC-RMA normalization method for the calculation [34]. After quantile normalization, the data were merged with the GNF1H-derived and GC-RMA normalized data (downloaded from Geneatlas website).

Notably, Rosetta1 and Rosetta2 does not include replicates of samples, whereas Stanford contains biological replicates and Geneatlas includes technical replicates by hybridizing the same tissue samples to two different Affymetrix chips. For comparative analysis, microarray probes were mapped to a common index system, in our case Entrez Gene Ids using the annotation provided by the authors or the SOURCE database http://smd.stanford.edu/cgi-bin/source/sourceSearch. Probes which could not be mapped were excluded from the analysis. Signals from probes linked to the same gene were averaged. In order to assess and to compare the tissue expression, the samples were assigned based on their physiology and histology to 19 main tissue classes found in all four data sets. The 19 main tissue classes are adrenal gland, brain, heart, kidney, liver, lung, lymph node, muscle, prostate, pancreas, placenta, salivary gland, thymus, thyroid, tonsil, testis, trachea, uterus, and uterine corpus. Expression values of tissues samples assigned to the same class were averaged to facilitate the comparison. Tables including the averaged expression values for the analysed data sets can be found as Additional files 2, 3, 4 and 5. Detailed information about the performed classification of tissues and the distribution of tissue across the different microarray experiments can be found in the Additional file 1 - Table S2.

### Correlation of correlations

Global similarity of expression patterns in different expression data sets can be assessed using 'correlation of correlations' [22]. This recently introduced measure is based on the comparison of pair-wise correlations of genes (or tissue samples, respectively) in the two data sets. For the gene-based comparison, Spearman correlation coefficients for each of the possible pairs of genes are calculated first for each data set (using only those genes found in the both data sets). The correlation coefficients obtained for the two data sets are subsequently correlated. Mathematically, this procedure can be defined as:

where *U*_*ij *_is the Spearman rank correlation of genes *i *and *j *in data set *U*, *V*_*ij *_is the Spearman rank correlation of genes *i *and *j *in data set *V*, and the sums are over all distinct pairs of genes *i *and *j*. Large correlation of correlations signifies similar co-expression of genes in two data sets. Similarly, we can proceed for tissue-based comparisons. The same steps as for the gene-based comparison were applied, but the Spearman correlation coefficients were calculated across all genes for all possible pairs of tissue types. For the tissue-based comparison, large correlation of correlations indicates similar tissue expression patterns.

The obtained results were used for hierarchical clustering to evaluate the similarity of expression patterns in the compared data sets. As distance measure, we chose 1-*r*_*UV*_. Clustering was conducted using Ward's minimum variance method and visualized with clustered image maps (heatmaps).

### Measures for tissue specific expression

We used the previously introduced Preferential Expression Measure (PEM) to identify genes that are over- or under-expressed in a specific tissue [13]. PEM describes the expression of a gene in a given tissue in relation to its average expression across all tissues. PEM reports a positive value for over-expressed genes and a negative value for under-expressed genes. For microarray experiments, the PEM for a tissue class *t*_*i *_can be defined as:

where *S *is the average expression of a gene in the specific tissue class *t*_*i *_and *A *is the arithmetic mean expression of the gene in all tissues (starting from log_2_-transformed expression values). To derive the significance of observed PEM scores, a permutation-based procedure was employed. Background distributions of PEM scores were generated based on repeated (N = 100) random association of the same number of arrays to a tissue class as observed for the original data set. Notably, this procedure conserves the number of arrays included in a specific tissue class. Subsequently, PEM scores were calculated separately for each tissue class and each data set. As measure for statistical significance, we used the false discovery rate (FDR). It is defined here as the expected proportion of false positives among all genes detected as tissue-specifically expressed. We can derive an empirical false discovery rate for a chosen PEM score *s*:

where *PEM*_*o *_and *PEM*_*b *_are the scores calculated for the original and randomized data sets, N is the number of randomization (N = 100) and δ(x) = 1 for x ≥ 0, respectively δ(x) = 0 for x < 0. Thus, the significance of the observed PEM scores can be derived by comparison with the generated background distributions [35]. For our analysis, genes with a positive PEM score achieving FDR < 0.25 were defined as tissue-specifically over-expressed whereas genes with negative PEM score and a FDR < 0.25 were defined as under-expressed.

The uniqueness of tissue-specific over-expression was evaluated by comparing the fold changes of the two tissues with the highest gene expression for each gene. We called this measure MAX, which can be defined as:

where *fc*(*t*) comprises the fold changes in all tissues *t *compared to the mean expression and *fc*(*t*') comprises the fold changes in all tissues except the one with the highest fold change of this gene, respectively. Note that this MAX score is assigned to a gene only for the tissue class with the largest expression; all other MAX scores of the gene are set to zero for the remaining tissue classes. A gene with a large MAX value shows high gene expression in one tissue but not in the other tissues, i.e. it is *uniquely *over-expressed. To assess the significance, the same permutation-based procedure as applied to PEM scores was employed. In our study, genes with a FDR < 0.25 were denoted as uniquely tissue-specifically over-expressed. The derived MAX and PEM scores as well as their corresponding FDR for different tissues and data sets can be found in Additional file 6 and 7. For the Geneatlas data set, we additionally provide the corresponding results for the alternative pre-processing and annotation scheme in Additional file 8.

The detected dependence between the number of tissue-specific genes and the number of samples included in a tissue class may interfere with a comparison among tissues. In order to reduce such interference, an adjustment of FDR thresholds for tissue-specific over-expression was performed for each data set independently. First, we calculated the number of genes *N *which were significantly over-expressed in brain in a chosen data set based on a FDR threshold of α (with α = 0.01, 0.05, 0.10 and 0.25). Second, a sub-set of *s *brain samples were randomly selected and the remaining non-selected brain samples were subsequently excluded from the data set. PEM scores for brain-specific over-expression and corresponding FDR were derived as previously described for this reduced data set. The FDR threshold for significant brain-specific over-expression was adjusted to a level α' so that the number of significant genes was the same as detected number *N *for the full set using a FDR threshold of α. The procedure was repeatedly performed (n = 10^3^) generating a distribution of adjusted thresholds. As a conserved estimate for adjustment of the FDR threshold, the 5^th ^percentile was chosen. The size *s *of the sub-set was set to values that reflected the number of observed samples in different tissue classes (given by Additional file 1 - table S2).

### Assessment of reliability by cross-validation

To assess the reliability of the consolidated gene lists we employed a cross-validation method. First of all, we reduced the number of genes to 6685, i.e. all the genes analyzed in all four data sets. Next, only those genes with MAX score > 0 for brain in all four data sets were selected (140 genes). For those genes we calculated the Spearman rank correlation between each of the four data sets. For cross-validation we consolidated three of the four data sets for each of the four possible combinations. For assessment of reliability we calculated the Spearman rank correlation of the consolidated gene lists and the gene list set not included in the consolidation and compared this value to the mean of the correlation values for the gene lists set based on individual data sets.

### GO analysis of over-expressed genes

We utilized gene annotation by Gene Ontology to assign tissue types to biological processes. Here, we assessed whether over-expressed genes in a given tissue type tend to be associated with specific biological processes. First, we calculated the enrichment of tissue-specific genes based with a PEM > 0.8 in biological process categories. The obtained scores for enrichment can subsequently be used to assign weighted profiles of biological processes to the given tissue type. A well-known difficulty, however, is the selection of the suitable set of GO categories, as many of categories are highly specific. Here, we aimed to select only those categories that include enough genes for adequate statistical validation but not too many genes to ensure the functional homogeneity of member genes. Following a previously proposed scheme, we chose therefore so-called *informative *GO categories: Each GO category should contain more than 300 genes and each of their child categories should contain less than 300 genes [36]. The significance of association between tissue type and GO category was calculated using Fisher's exact test assessing the accumulation of over-expressed genes in a tested category. P-values obtained were adjusted and converted to false discovery rates (FDRs) using the Benjamini-Hochberg method [37]. A small FDR signifies enrichment of tissue-specifically over-expressed genes in a GO category. To examine the similarities of enrichment profiles of the different tissue types, the results were visualized as cluster image maps.

## Authors' contributions

JR assembled the data sets, performed the comparative analysis and prepared the original draft of the manuscript. MF conceived of the study and guided the gene expression analysis and the interpretation of the results. Both authors read and approved the final manuscript.

## Supplementary Material

Additional file 1 Supplementary materials including tables and figures (pdf). Click here for file

Additional file 2 Expression values of Rosetta1 data set after merging to the 19 main tissue classes (tab-delimited table). Click here for file

Additional file 3 Expression values of Rosetta2 data set after merging to the 19 main tissue classes (tab-delimited table). Click here for file

Additional file 4 Expression values of Geneatlas data set after merging to the 19 main tissue classes (tab-delimited table). Click here for file

Additional file 5 Expression values of Stanford data set after merging to the 19 main tissue classes (tab-delimited table). Click here for file

Additional file 6 MAX scores derived for the different expression data sets and tissue classes and the corresponding FDR for the 6685 non-redundant genes included in the comparison (tab-delimited table). Click here for file

Additional file 7 PEM scores derived for the different expression data sets and tissue classes and the corresponding FDR for the 6685 non-redundant genes included in the comparison (tab-delimited table). Click here for file

Additional file 8 MAX scores and corresponding FDR derived for the Geneatlas data set after application of alternative annotation and normalization (tab-delimited table). Click here for file

## References

[B1] Alizadeh AA Eisen MB Davis RE Ma C Lossos IS Rosenwald A Boldrick JC Sabet H Tran T Yu X Distinct types of diffuse large B-cell lymphoma identified by gene expression profiling Nature 2000 403 502 511 10.1038/35000501 10676951

[B2] Golub TR Slonim DK Tamayo P Huard C Gaasenbeek M Mesirov JP Coller H Loh ML Downing JR Caligiuri MA Molecular classification of cancer: class discovery and class prediction by gene expression monitoring Science 1999 286 531 537 10.1126/science.286.5439.531 10521349

[B3] Futschik ME Sullivan M Reeve A Kasabov N Prediction of clinical behaviour and treatment for cancers Applied bioinformatics 2003 2 3 Suppl S53 58 15130817

[B4] Rosenwald A Wright G Chan WC Connors JM Campo E Fisher RI Gascoyne RD Muller-Hermelink HK Smeland EB Giltnane JM The use of molecular profiling to predict survival after chemotherapy for diffuse large-B-cell lymphoma N Engl J Med 2002 346 1937 1947 10.1056/NEJMoa012914 12075054

[B5] Johnson JM Castle J Garrett-Engele P Kan Z Loerch PM Armour CD Santos R Schadt EE Stoughton R Shoemaker DD Genome-wide survey of human alternative pre-mRNA splicing with exon junction microarrays Science 2003 302 2141 2144 10.1126/science.1090100 14684825

[B6] Saito-Hisaminato A Katagiri T Kakiuchi S Nakamura T Tsunoda T Nakamura Y Genome-wide profiling of gene expression in 29 normal human tissues with a cDNA microarray DNA Research 2002 9 35 45 10.1093/dnares/9.2.35 12056413

[B7] Su AI Cooke MP Ching KA Hakak Y Walker JR Wiltshire T Orth AP Vega RG Saphinoso LM Moqrich A Large-scale analysis of the human and mouse transcriptomes Proc Natl Acad Sci USA 2002 99 7 4465 4470 10.1073/pnas.012025199 11904358 PMC123671

[B8] Su AI Wiltshire T Batalov S Lapp H Ching KA Block D Zhang J Soden R Hayakawa M Kreiman G A gene atlas of the mouse and human protein-encoding transcriptomes Proc Natl Acad Sci USA 2004 101 16 6062 6067 10.1073/pnas.0400782101 15075390 PMC395923

[B9] Kuo WP Jenssen TK Butte AJ Ohno-Machado L Kohane IS Analysis of matched mRNA measurements from two different microarray technologies Bioinformatics 2002 18 3 405 412 10.1093/bioinformatics/18.3.405 11934739

[B10] Michiels S Koscielny S Hill C Prediction of cancer outcome with microarrays: a multiple random validation strategy Lancet 2005 365 488 492 10.1016/S0140-6736(05)17866-0 15705458

[B11] Miklos GLG Maleszka R Microarray reality checks in the context of a complex disease Nat Biotechnology 2004 22 615 621 10.1038/nbt965 15122300

[B12] Tan PK Downey TJ Spitznagel ELJ Xu P Fu D Dimitrov DS Lempicki RA Raaka BM Cam MC Evaluation of gene expression measurements from commercial microarray platforms Nucleic Acids Res 2003 31 5676 5684 10.1093/nar/gkg763 14500831 PMC206463

[B13] Huminiecki L Lloyd AT Wolfe KH Congruence of tissue expression profiles from Gene Expression Atlas, SAGEmap and TissueInfo databases BMC Genomics 2003 4 31 10.1186/1471-2164-4-31 12885301 PMC183867

[B14] Rhodes DR Yu J Shanker K Deshpande N Varambally R Ghosh D Barrette T Pandey A Chinnaiyan AM Large-scale meta-analysis of cancer microarray data identifies common transcriptional profiles of neoplastic transformation and progression Proc Natl Acad Sci USA 2004 101 25 9309 9314 10.1073/pnas.0401994101 15184677 PMC438973

[B15] Boyer LA Lee TI Cole MF Johnstone SE Levine SS Zucker JP Guenther MG Kumar RM Murray HL Jenner RG Core transcriptional regulatory circuitry in human embryonic stem cells Cell 2005 122 947 956 10.1016/j.cell.2005.08.020 16153702 PMC3006442

[B16] Futschik ME Chaurasia G Tschaut A Russ J Babu MM Herzel H Functional and transcriptional coherency of modules in the human protein interaction network Journal of Integrative Bioinformatics 2007 4 3 76 10.2390/biecoll-jib-2007-76

[B17] Juric D Sale S Hromas RA Yu R Wang Y Duran GE Tibshirani R Einhorn LH Sikic BI Gene expression profiling differentiates germ cell tumors from other cancers and defines subtype-specific signatures Proc Natl Acad Sci USA 2005 102 17763 17768 10.1073/pnas.0509082102 16306258 PMC1308932

[B18] Yanai I Korbel JO Boue S McWeeney SK Bork P Lercher MJ Similar gene expression profiles do not imply similar tissue functions Trends in Genetics 2006 22 132 138 10.1016/j.tig.2006.01.006 16480787

[B19] Young JI Hong EP Castle JC Crespo-Barreto J Bowman AB Rose MF Kang D Richman R Johnson JM Berget S Regulation of RNA splicing by the methylation-dependent transcriptional repressor methyl-CpG binding protein 2 Proc Natl Acad Sci USA 2005 102 17551 17558 10.1073/pnas.0507856102 16251272 PMC1266160

[B20] Schadt EE Edwards SW GuhaThakurta D Holder D Ying L Svetnik V Leonardson A Hart KW Russell A Li G A comprehensive transcript index of the human genome generated using microarrays and computational approaches Genome Biology 2004 5 R73 10.1186/gb-2004-5-10-r73 15461792 PMC545593

[B21] Shyamsundar R Kim YH Higgins JP Montgomery K Jorden M Sethuraman A Rijn M van de Botstein D Brown PO Pollack JR A DNA microarray survey of gene expression in normal human tissues Genome Biology 2005 6 R22 10.1186/gb-2005-6-3-r22 15774023 PMC1088941

[B22] Lee JK Bussey KJ Gwadry FG Reinhold W Riddick G Pelletier SL Nishizuka S Szakacs G Annereau J-P Shankavaram U Comparing cDNA and oligonucleotide array data: concordance of gene expression across platforms for the NCI-60 cancer cells Genome Biology 2003 4 R82 10.1186/gb-2003-4-12-r82 14659019 PMC329421

[B23] Brenner M Johnson AB Boespflug-Tanguy O Rodriguez D Goldman JE Messing A Mutations in GFAP, encoding glial fibrillary acidic protein, are associated with Alexander disease Nature genetics 2001 27 1 117 120 10.1038/83679 11138011

[B24] Schmechel D Marangos PJ Zis AP Brightman M Goodwin FK Brain endolases as specific markers of neuronal and glial cells Science 1978 199 4326 313 315 10.1126/science.339349 339349

[B25] Delanghe JR Langlois MR Hemopexin: a review of biological aspects and the role in laboratory medicine Clinica chimica acta; international journal of clinical chemistry 2001 312 1-2 13 23 10.1016/S0009-8981(01)00586-1 11580905

[B26] Carter SL Eklund AC Mecham BH Kohane IS Szallasi Z Redefinition of Affymetrix probe sets by sequence overlap with cDNA microarray probes reduces cross-platform inconsistencies in cancer-associated gene expression measurements BMC bioinformatics 2005 6 107 10.1186/1471-2105-6-107 15850491 PMC1127107

[B27] Neerincx PB Casel P Prickett D Nie H Watson M Leunissen JA Groenen MA Klopp C Comparison of three microarray probe annotation pipelines: differences in strategies and their effect on downstream analysis BMC proceedings 2009 3 Suppl 4 S1 10.1186/1753-6561-3-s4-s1 19615109 PMC2712739

[B28] Larkin JE Frank BC Gavras H Sultana R Quackenbush J Independence and reproducibility across microarray platforms Nature methods 2005 2 5 337 344 10.1038/nmeth757 15846360

[B29] Basso K Margolin AA Stolovitzky G Klein U Dalla-Favera R Califano A Reverse engineering of regulatory networks in human B cells Nature genetics 2005 37 4 382 390 10.1038/ng1532 15778709

[B30] Chaurasia G Malhotra S Russ J Schnoegl S Hanig C Wanker EE Futschik ME UniHI 4: new tools for query, analysis and visualization of the human protein-protein interactome Nucleic Acids Res 2009 37 Database D657 660 10.1093/nar/gkn841 18984619 PMC2686569

[B31] Bolstad BM Irizarry RA Astrand M Speed TP A comparison of normalization methods for high density oligonucleotide array data based on variance and bias Bioinformatics 2003 19 2 185 193 10.1093/bioinformatics/19.2.185 12538238

[B32] Futschik ME Crompton T Model selection and efficiency testing for normalization of cDNA microarray data Genome Biology 2004 5 R60 10.1186/gb-2004-5-8-r60 15287982 PMC507885

[B33] Futschik ME Crompton T OLIN: optimized normalization, visualization and quality testing of two-channel microarray data Bioinformatics 2005 21 1724 1726 10.1093/bioinformatics/bti199 15585527

[B34] Ferrari F Bortoluzzi S Coppe A Sirota A Safran M Shmoish M Ferrari S Lancet D Danieli GA Bicciato S Novel definition files for human GeneChips based on GeneAnnot BMC Bioinformatics 2007 8 446 10.1186/1471-2105-8-446 18005434 PMC2216044

[B35] Futschik ME Herzel H Are we overestimating the number of cell-cycling genes? The impact of background models on time-series analysis Bioinformatics 2008 24 8 1063 1069 10.1093/bioinformatics/btn072 18310054

[B36] Zhou X Kao M-CJ Wong WH Transitive functional annotation by shortest path analysis of gene expression data Proc Natl Acad Sci USA 2002 99 12783 12788 10.1073/pnas.192159399 12196633 PMC130537

[B37] Efron B Tibshirani R Empirical bayes methods and false discovery rates for microarrays Genet Epidemiol 2002 23 70 86 10.1002/gepi.1124 12112249

