# Supplementary material for: Comparison and consolidation of microarray data sets of human tissue expression
Source: BMC Genomics. 2010 May 14;11:305. doi: 10.1186/1471-2164-11-305 (PMC2885367; doi:10.1186/1471-2164-11-305)

Supplementary materials

Comparison and consolidation of microarray data sets of human tissue expression

Jenny Russ and Matthias E. Futschik

Institute for Theoretical Biology, Charité, Humboldt University, Germany and
Centre for Molecular and Structural Biomedicine, University of Algarve, Portugal

Table S1 – Tissue-wise correlation of correlations

Correlation of tissue-wise correlations between the four data sets and between corresponding randomized gene expression matrices.

		**Rosetta1**	**Rosetta2**	**Geneatlas**	**Stanford**
**Rosetta1**	**Original**	**1**	**0.67**	**0.49**	**0.50**
	Random	0.19	0.22	0.16	0.17
**Rosetta2**	**Original**	**0.67**	**1**	**0.46**	**0.53**
	Random	0.22	0.04	0.22	0.27
**Geneatlas**	**Original**	**0.49**	**0.46**	**1**	**0.37**
	Random	0.16	0.22	0.13	0.12
**Stanford**	**Original**	**0.50**	**0.53**	**0.37**	**1**
	Random	0.17	0.27	0.12	0.07

Table S2 – Number of samples in tissue classes

Number of samples assigned to the 19 main tissue classes for the four microarray experiments

Tissue class	Rosetta1	Rosetta2	Geneatlas	Stanford
**Adrenal gland**	2	3	4	4
**Brain**	10	9	44	8
**Heart**	1	1	6	7
**Kidney**	2	2	2	5
**Liver**	2	2	4	5
**Lung**	2	2	4	4
**Lymph node**	1	1	2	5
**Muscle**	1	1	3	3
**Prostate**	1	1	2	5
**Placenta**	1	1	2	1
**Pancreas**	1	1	4	2
**Salivary gland**	1	1	2	4
**Thymus**	1	1	2	2
**Thyroid**	1	1	4	7
**Tonsil**	1	1	2	4
**Testis**	1	1	10	7
**Trachea**	1	1	2	2
**Uterus**	1	1	2	4
**Uterine corpus**	1	1	2	1

The tissue classes were composed of following tissue samples found in the compared tissue expression data sets*:* ***Adrenal gland*** - adrenal gland, adrenal medullar, adrenal cortex; ***Brain*** - whole brain, temporal lobe, globus pallidus, cerebellum peduncles, cerebellum, caudate nucleus, parietal lobe, medulla oblongata, amygdale, prefrontal cortex, occipital lobe, hypothalamus, thalamus, subthalamic nucleus, cingulated cortex, pons, fetal brain, ciliary ganglion, trigeminal ganglion, dorsal root ganglion, superior cervical ganglion, olfactory bulb, frontal cortex, occipital cortex, temporal cortex, brain, cerebral cortex, corpus callosum, hippocampus, postcentral gyrus, ***Heart*** - heart, cardiac myocytes, atrioventricular node, pericardium, ***Kidney*** - kidney, fetal kidney, ***Liver*** - liver, fetal liver, ***Lung*** - lung, fetal lung, human bronchial epithelial cells, ***Lymph node*** - lymph node, ***Muscle*** - skeletal muscle, smooth muscle, diaphragm, muscle, ***Prostate*** - prostate, ***Placenta*** - placenta, ***Pancreas*** - pancreas, pancreatic islet, ***Salivary gland*** - salivary gland, ***Thymus*** - thymus, ***Thyroid*** - thyroid, fetal thyroid, ***Tonsil*** - tonsil, ***Testis*** - testis, leydig cell, germ cell, interstitial, seminiferous tubule, seminal vesicle, epididymus, ***Trachea*** - trachea, ***Uterus*** - uterus and ***Uterine corpus*** - uterine corpus.

Table S3 – Gene-wise correlation of correlation for alternatively pre-processed data sets

Correlation of gene-wise correlations between the alternatively pre-processed (see *Methods* section for details) data sets and between corresponding randomized gene expression matrices.

		**Rosetta1**	**Rosetta2**	**Geneatlas**	**Stanford**
**Rosetta1**	**Original**	**1**	**0.57**	**0.30**	**0.18**
	Random	0.0027	0.0030	0.0041	0.0050
**Rosetta2**	**Original**	**0.57**	**1**	**0.28**	**0.20**
	Random	0.0030	0.0021	0.0028	0.0037
**Geneatlas**	**Original**	**0.30**	**0.28**	**1**	**0.14**
	Random	0.0041	0.0028	0.0035	0.0055
**Stanford**	**Original**	**0.18**	**0.20**	**0.14**	**1**
	Random	0.0050	0.0037	0.0055	0.0027

Table S4 – Comparison of original and alternative annotation and pre-processing methods for Geneatlas data set

Number of genes detected as tissue-specially over-expressed using the MAX score in the Geneatlas data set after application of different pre-processing and annotation schemes (see main text) are shown for the tissue included in the platform comparison. Also, the number of common tissue-specific genes detected for both schemes as well as the corresponding significance of the observed overlap is shown. For the comparison, genes were mapped to Entrez Gene IDs. In total, 14848 genes were common to both annotation schemes. The significance was calculated using the hypergeometric distribution. Notably, the overlap is highly significant for all compared tissues.

Tissue	Original pre-processing & annotation	Alternative pre-processing & annotation	Number of common genes	Significance of overlap
**Adrenal gland**	682	904	586	$p = 2.72 \cdot 10^{-127}$
**Brain**	67	87	57	$p < 1.00 \cdot 10^{-300}$
**Kidney**	34	59	28	$p = 3.37 \cdot 10^{-65}$
**Liver**	419	393	275	$p < 1.00 \cdot 10^{-300}$
**Lung**	61	105	39	$p = 7.22 \cdot 10^{-72}$

Table S5 – Adjusted FDR thresholds for tissue-specific over-expression

Adjusted FDR thresholds are shown which correspond to selected FDR thresholds for brain-specific over-expression in the different data sets. The selected FDR thresholds for brain-specific over-expression are 0.01 in (A), 0.05 in (B), 0.10 (C) and 0.25 (D).

A) FDR (Brain) = 0.01

Tissue class	Rosetta1	Rosetta2	Geneatlas	Stanford
**Adrenal gland**	0.14	0.07	0.14	0.03
**Brain**	0.01	0.01	0.01	0.01
**Heart**	-	-	0.10	0.02
**Kidney**	0.14	0.15	0.28	0.02
**Liver**	0.14	0.15	0.14	0.02
**Lung**	0.14	0.15	0.14	0.03
**Lymph node**	-	-	0.28	0.02
**Muscle**	-	-	0.20	0.06
**Prostate**	-	-	0.28	0.02
**Placenta**	-	-	0.28	-
**Pancreas**	-	-	0.14	0.15
**Salivary gland**	-	-	0.28	0.03
**Thymus**	-	-	0.28	0.15
**Thyroid**	-	-	0.14	0.02
**Tonsil**	-	-	0.28	0.03
**Testis**	-	-	0.05	0.02
**Trachea**	-	-	0.28	0.15
**Uterus**	-	-	0.28	0.03
**Uterine corpus**	-	-	0.28	-

B) FDR (Brain) = 0.05

Tissue class	Rosetta1	Rosetta2	Geneatlas	Stanford
**Adrenal gland**	0.24	0.16	0.24	0.11
**Brain**	0.05	0.05	0.05	0.05
**Heart**	-	-	0.19	0.06
**Kidney**	0.24	0.25	0.38	0.08
**Liver**	0.24	0.25	0.24	0.08
**Lung**	0.24	0.25	0.24	0.11
**Lymph node**	-	-	0.38	0.08
**Muscle**	-	-	0.30	0.18
**Prostate**	-	-	0.38	0.08
**Placenta**	-	-	0.28	-
**Pancreas**	-	-	0.24	0.30
**Salivary gland**	-	-	0.38	0.11
**Thymus**	-	-	0.38	0.30
**Thyroid**	-	-	0.24	0.08
**Tonsil**	-	-	0.38	0.11
**Testis**	-	-	0.12	0.08
**Trachea**	-	-	0.38	0.30
**Uterus**	-	-	0.38	0.11
**Uterine corpus**	-	-	0.38	-

C) FDR (Brain) = 0.10

Tissue class	Rosetta1	Rosetta2	Geneatlas	Stanford
**Adrenal gland**	0.31	0.30	0.30	0.23
**Brain**	0.10	0.10	0.10	0.10
**Heart**	-	-	0.25	0.16
**Kidney**	0.31	0.34	0.42	0.17
**Liver**	0.31	0.34	0.30	0.17
**Lung**	0.31	0.34	0.30	0.23
**Lymph node**	-	-	0.42	0.17
**Muscle**	-	-	0.35	0.34
**Prostate**	-	-	0.42	0.17
**Placenta**	-	-	0.42	-
**Pancreas**	-	-	0.30	0.50
**Salivary gland**	-	-	0.42	0.23
**Thymus**	-	-	0.42	0.50
**Thyroid**	-	-	0.30	0.16
**Tonsil**	-	-	0.42	0.23
**Testis**	-	-	0.17	0.16
**Trachea**	-	-	0.42	0.50
**Uterus**	-	-	0.42	0.23
**Uterine corpus**	-	-	0.42	-

D) FDR (Brain) = 0.25

Tissue class	Rosetta1	Rosetta2	Geneatlas	Stanford
**Adrenal gland**	0.40	0.43	0.42	0.33
**Brain**	0.25	0.25	0.25	0.25
**Heart**	-	-	0.41	0.27
**Kidney**	0.40	0.53	0.42	0.28
**Liver**	0.40	0.53	0.42	0.28
**Lung**	0.40	0.53	0.53	0.33
**Lymph node**	-	-	0.53	0.28
**Muscle**	-	-	0.48	0.44
**Prostate**	-	-	0.53	0.28
**Placenta**	-	-	0.53	-
**Pancreas**	-	-	0.42	0.60
**Salivary gland**	-	-	0.42	0.33
**Thymus**	-	-	0.42	0.50
**Thyroid**	-	-	0.30	0.27
**Tonsil**	-	-	0.53	0.33
**Testis**	-	-	0.33	0.27
**Trachea**	-	-	0.53	0.60
**Uterus**	-	-	0.53	0.33
**Uterine corpus**	-	-	0.53	-

Figure S1 – Number of uniquely over-expressed genes

Number of uniquely over-expressed genes found in each data set and the corresponding overlap for different cut off of FDR. Only the tissue classes (adrenal gland, brain, kidney, liver and lung) for which the statistical significance could be derived were considered.

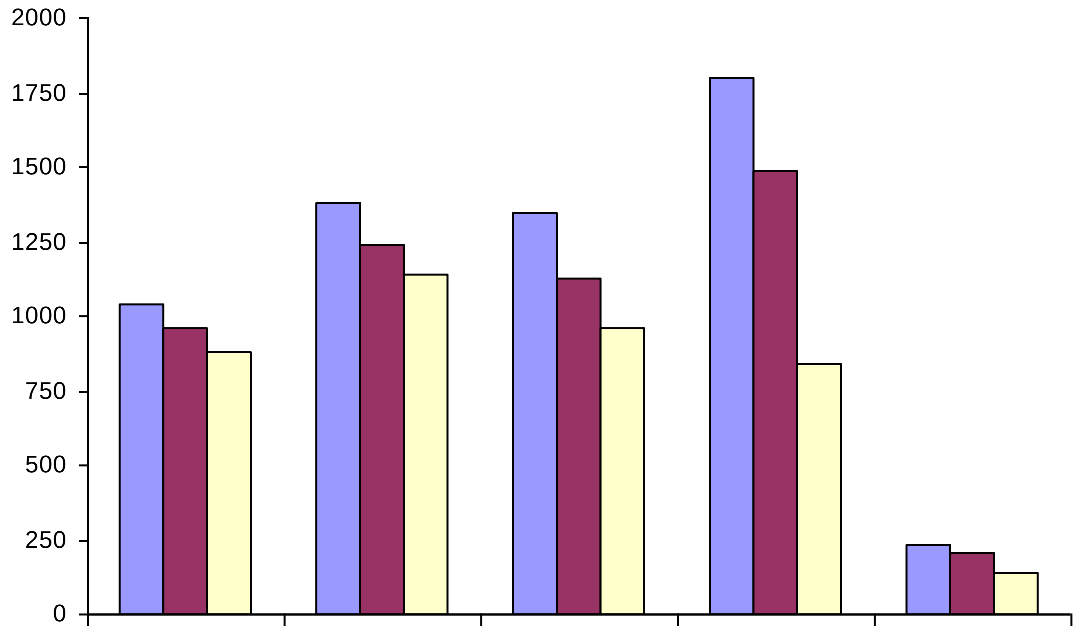

	Rosetta1	Rosetta2	Geneatlas	Stanford	Total Overlap
FDR < 0.25	1037	1383	1350	1801	232
FDR < 0.15	962	1240	1126	1490	205
FDR < 0.05	881	1137	963	841	143

Figure S2 – Number of tissue-specifically over-expressed genes detected consistently in all four data sets

Genes were defined as specifically over-expressed for PEM > 0 and FDR < 0.25. The bar-plot shows the number of genes fulfilling these criteria in all four datasets for the four tissue classes in which significant genes could be identified in all four data sets.

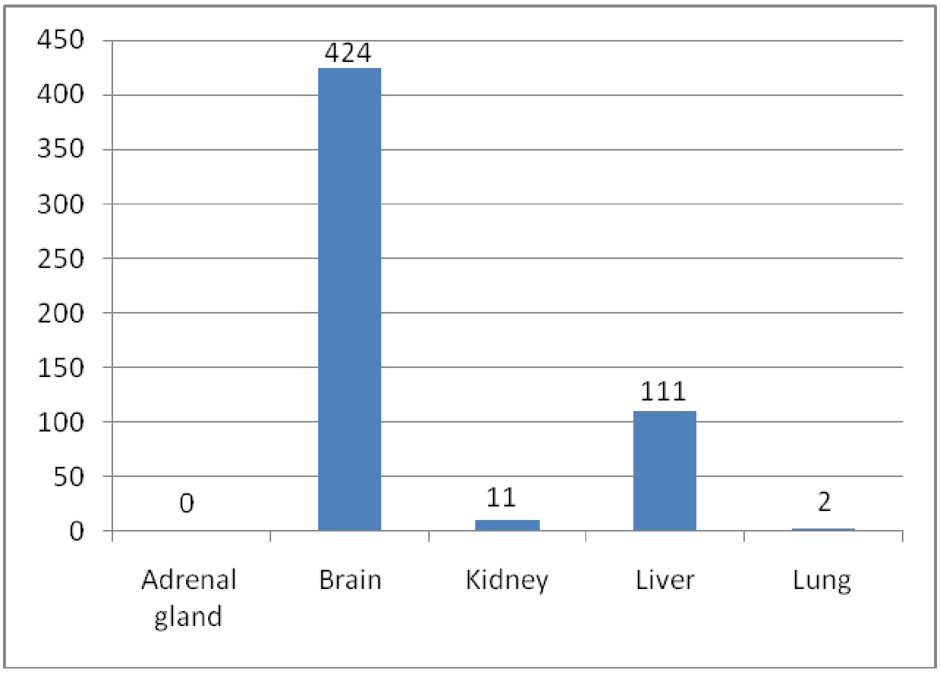

Figure S3 – Comparison of gene expression in brain and non-brain tissue for alternatively pre-processed data sets

Differential expression between brain and non-brain tissues was assessed by performing a gene-wise unpaired Student's t-test. To compare the results from different data sets, t-scores derived from each data set were plotted versus those from the other data sets for the corresponding genes. Additionally, the Pearson correlation coefficient is given.

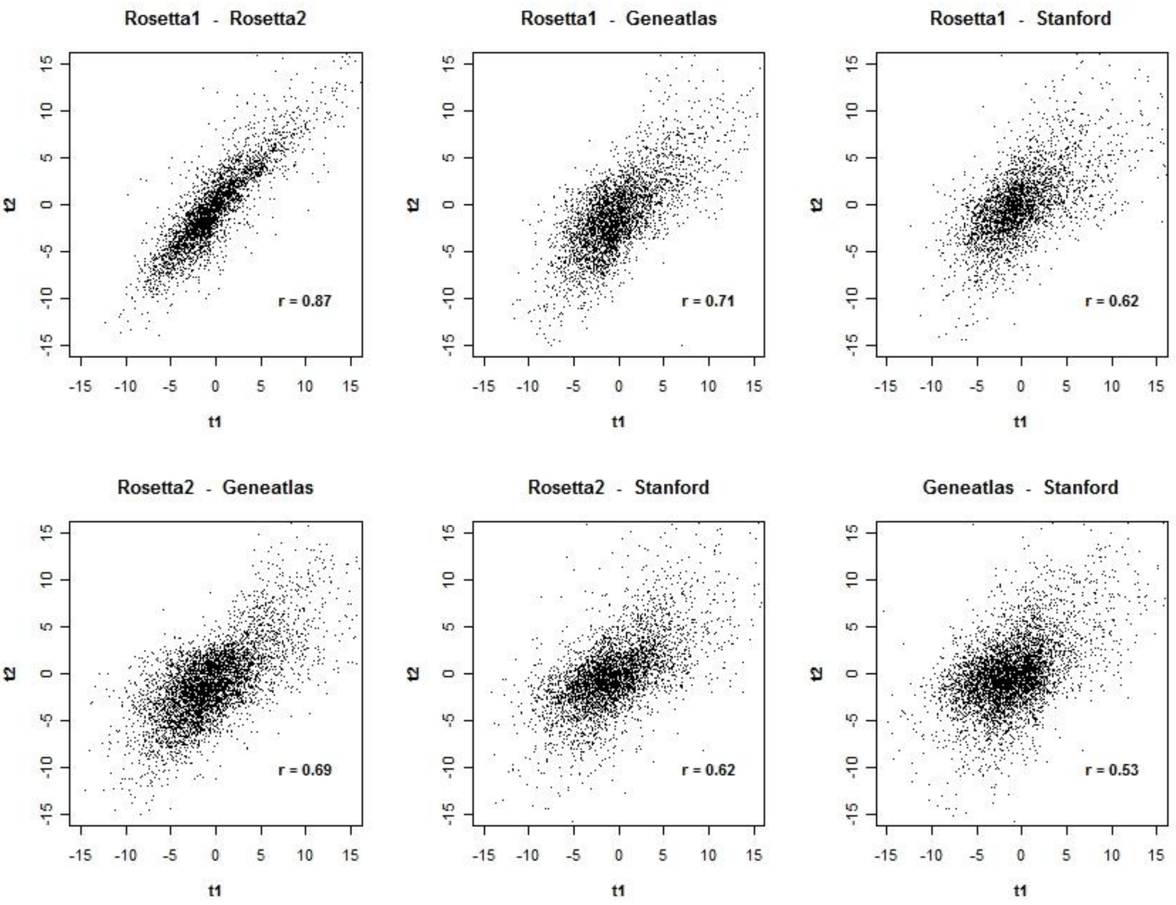

Figure S4 – Overlap of assayed and uniquely over-expressed genes for alternatively pre-processed data sets

A) The Venn-Diagram displays the overlap of all assayed genes in the different microarray experiments that were preprocessed using the alternative pre-processing scheme described in the *Methods* section. B) The overlap between the lists of uniquely over-expressed genes (defined by a positive MAX and a FDR < 0.25) derived from the four data sets are shown. Notably, the numbers of shared genes are similar to those determined for the original data sets indicating a limited impact of the choice of pre-processing on the results of the comparison.

A)

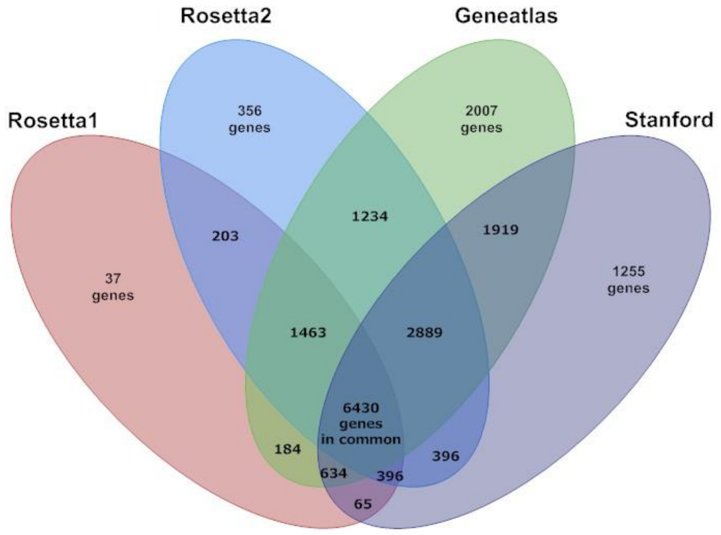

B)

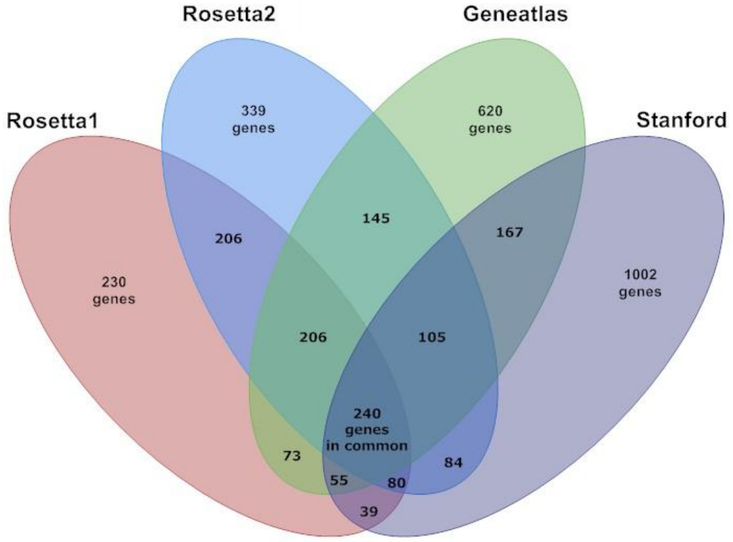

Figure S5 – Comparison of differential gene expression in brain for the Geneatlas data after application of alternative pre-processing and annotation schemes

Differential expression between brain and non-brain tissues was assessed by performing a gene-wise unpaired Student's t-test for the Geneatlas data set after application of the two pre-processing and annotation schemes described in the main text. For assessment of the influence of the pre-processing and annotation schemes, the resulting t-scores of the corresponding genes were plotted versus each other. A Pearson correlation coefficient of 0.87 was calculated (corresponding to a significance of $p < 10^{-16}$). Similar results were obtained for the differential expression in other tissues (data not shown).

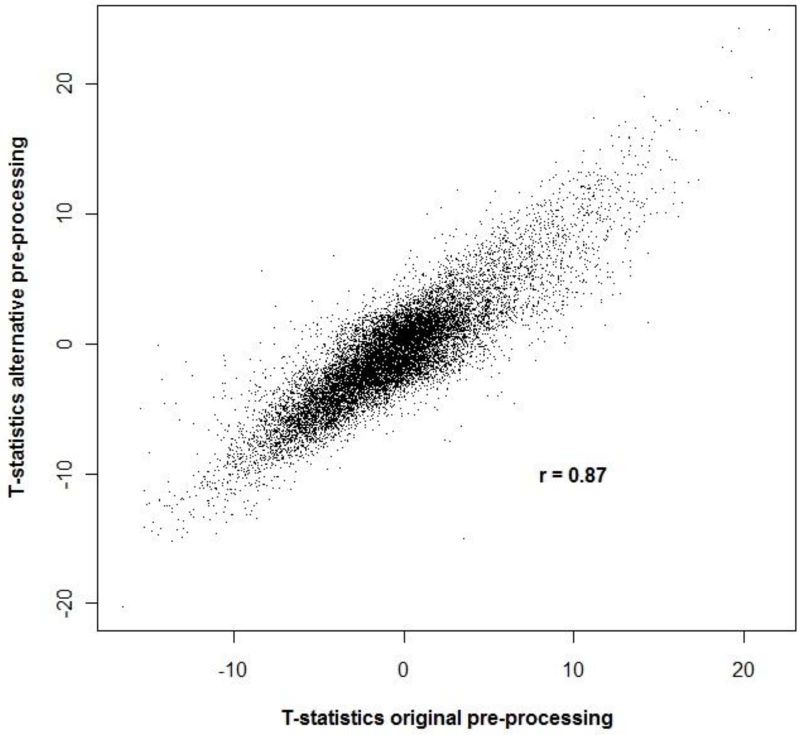

Figure S6 – Dependence of detected brain-specific genes on number of brain samples.

The plot shows the average number of genes that were detected as over-expressed in the (reduced) Geneatlas data set based on calculated PEM scores and a fixed threshold of FDR < 0.25. PEM scores and corresponding FDR were calculated for different number of sub-samples (n= 2, 3, 4, 6, 10) which were randomly drawn from the set of original 44 brain samples included in the data set. For each shown data point, 1000 random samples were generated and evaluated. Notably, a smaller number of samples lead to a strong decrease in the number of genes detected as specifically over-expressed in brain. Similar dependencies were observed for the other expression sets (data not shown).

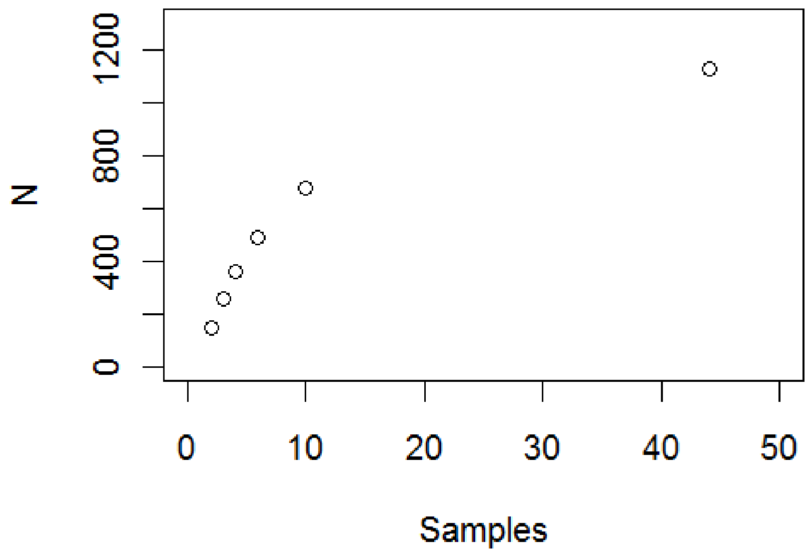

Figure S7 – Number of tissue-specifically over-expressed genes based on adjusted FDR thresholds.

The bar plots display the number of genes that were detected as significantly over-expressed in a specific tissue within the Rosetta1 (A), Rosetta2 (B), Genealtas (C) and Stanford data set (D). To detect tissue-specific over-expression, PEM scores and their corresponding FDR were calculated. The black shaded bars symbolize the number of genes detected as significant based on a fixed threshold of FDR < 0.05. In contrast, the gray shaded bars symbolize the number of additional genes if the FDR threshold is adjusted as described in the Methods sections. The applied FDR thresholds can be found in table S5B. Following abbreviations were used: AG - adrenal gland, B - brain, H - heart, K - kidney, LI -liver, LU - lung, LY - lymph node, M - muscle, PR - prostate, PA - pancreas, PL - placenta, SG - salivary gland, THYR - thyroid, THYM – thymus, TO - tonsil, TE - testis, TR - trachea, UT - uterus, and UC -uterine corpus.

A)

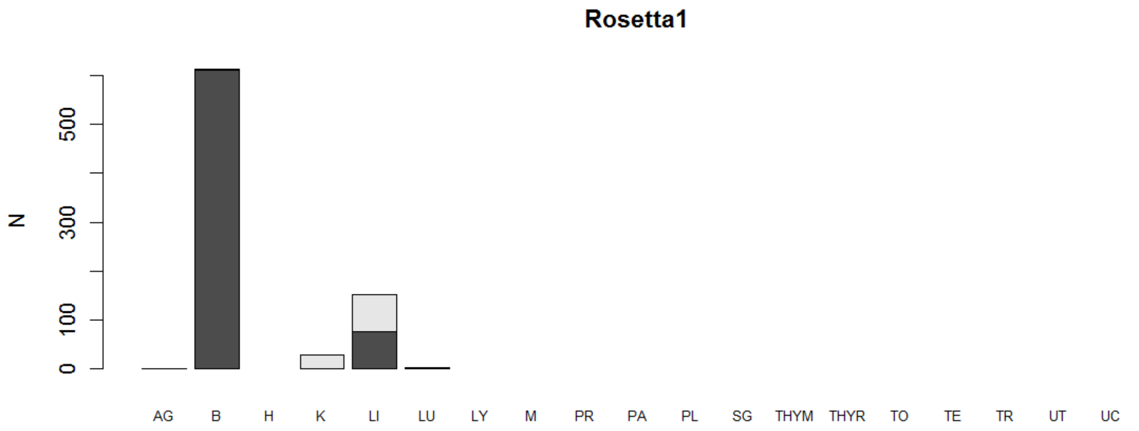

B)

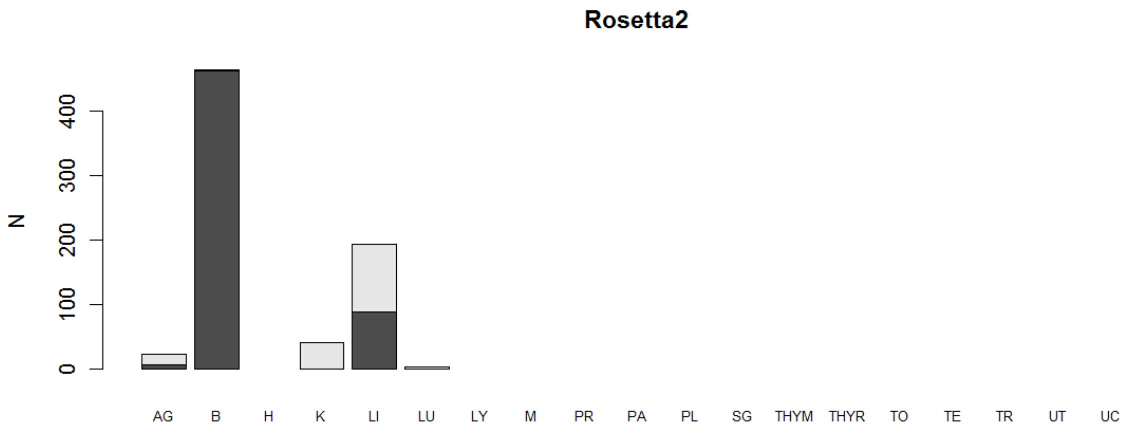

C)

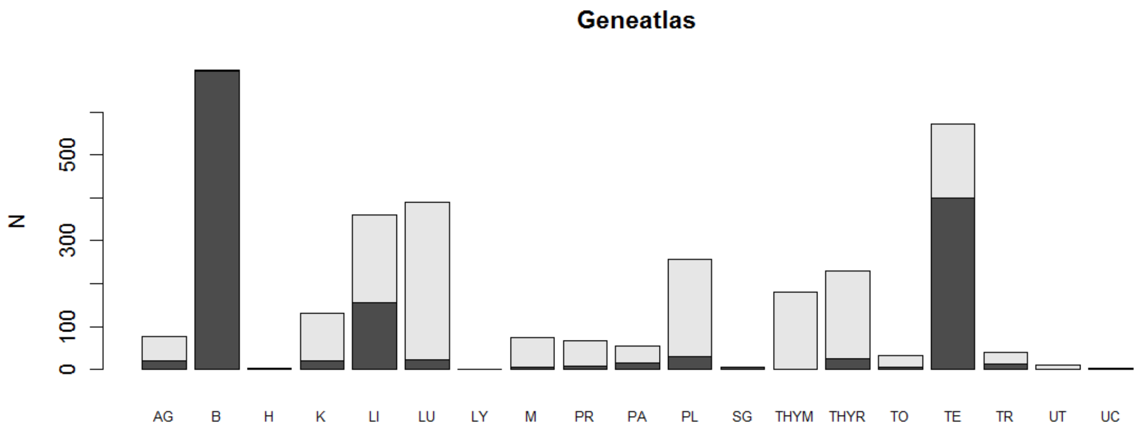
Geneatlas
N
0
100
300
500
AG
B
H
K
LI
LU
LY
M
PR
PA
PL
SG
THYM
THYR
TO
TE
TR
UT
UC

D)

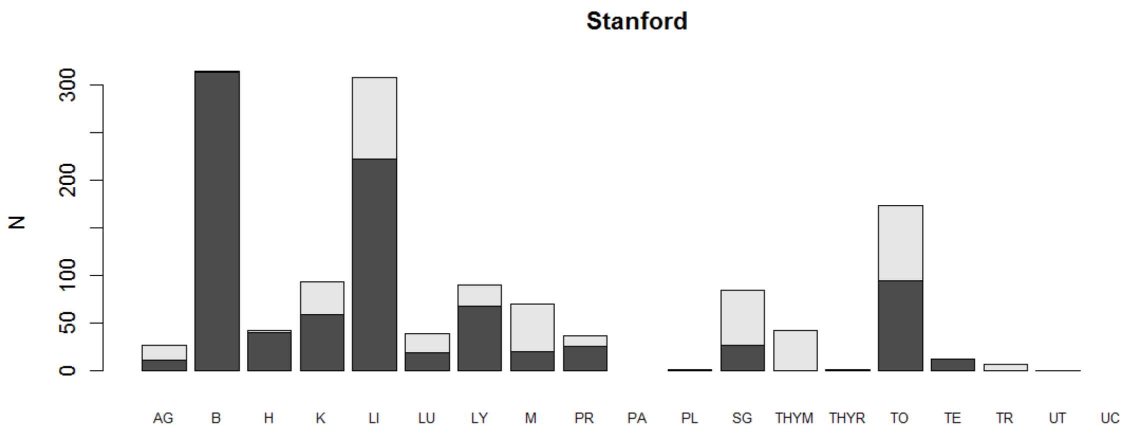
Stanford
N
0
50
100
200
300
AG
B
H
K
LI
LU
LY
M
PR
PA
PL
SG
THYM
THYR
TO
TE
TR
UT
UC

Figure S8 – Cluster image map for the GO analysis of the Rosetta1, Rosetta2 and Stanford data sets

Genes of the three data sets were mapped to the biological processes to which the genes are assigned in Gene Ontology (GO). The significance of enrichment in informative GO categories was derived by using Fisher's exact test and adjusted for multiple testing. Hierarchical clustering was subsequently performed based on the derived false discovery rates (FDR). The cluster image maps display the FDR of the GO enrichment according to the color-bar at the bottom.

A) Rosetta1

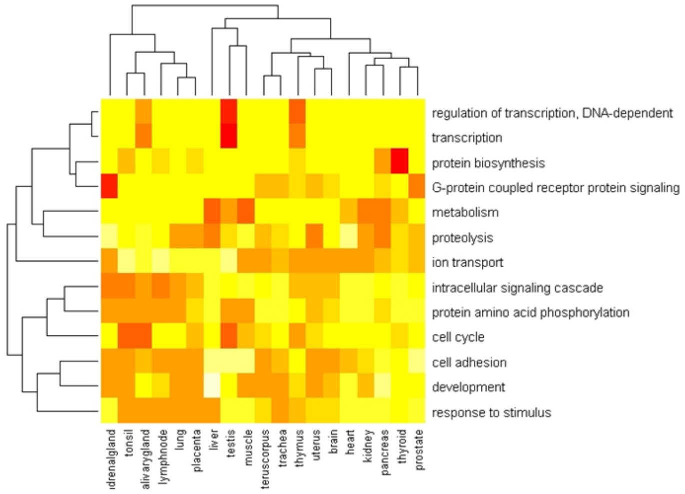

B) Rosetta2

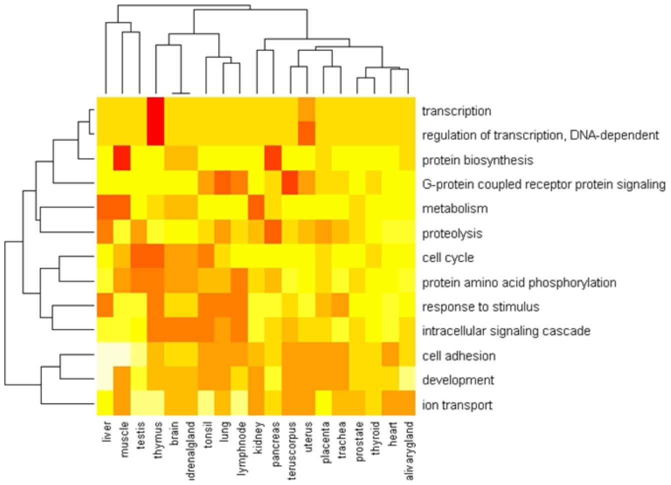

C) Stanford

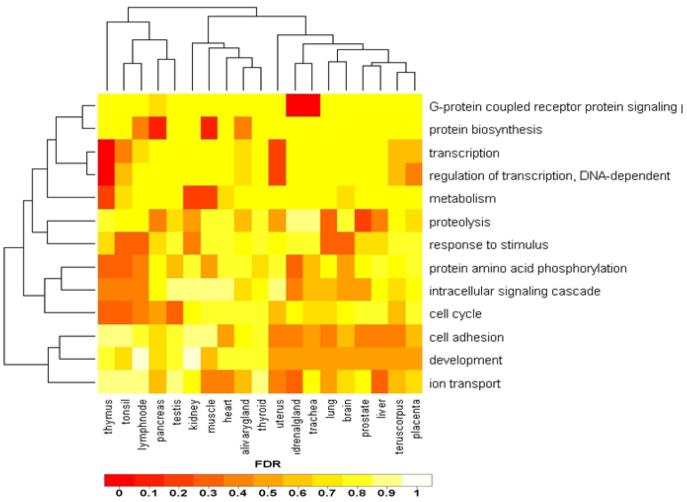

Supplement: Additional file 1 — Supplementary materials including tables and figures (pdf). [file 1471-2164-11-305-S1.PDF]
